# PhUGT78A22, a novel glycosyltransferase in *Paeonia* ‘He Xie’, can catalyze the transfer of glucose to glucosylated anthocyanins during petal blotch formation

**DOI:** 10.1186/s12870-022-03777-5

**Published:** 2022-08-18

**Authors:** Yang Li, Fan Kong, Zheng’an Liu, Liping Peng, Qingyan Shu

**Affiliations:** 1 Key Laboratory of Plant Resources and Beijing Botanical Garden, Institute of Botany, the Chinese Academy of Sciences, Beijing, 100093 China; 2 University of the Chinese Academy of Sciences, Beijing, 100049 China

**Keywords:** Anthocyanins di-glucosides, UDP-glycosyltransferase, Petal blotch formation, *Paeonia*, Anthocyanin distribution pattern

## Abstract

**Background:**

Flower color patterns play an important role in the evolution and subsequent diversification of flowers by attracting animal pollinators. This interaction can drive the diversity observed in angiosperms today in many plant families such as Liliaceae, Paeoniaceae, and Orchidaceae, and increased their ornamental values. However, the molecular mechanism underlying the differential distribution of anthocyanins within petals remains unclear in *Paeonia*.

**Results:**

In this study, we used an intersectional hybrid between the section *Moutan* and *Paeonia*, hereafter named *Paeonia* ‘He Xie’, which has purple flowers with dark purple blotches. After Ultra-high performance liquid chromatography-diode array detector (UPLC-DAD) analysis of blotched and non-blotched parts of petals, we found the anthocyanin content in the blotched part was always higher than that in the non-blotched part. Four kinds of anthocyanins, namely cyanidin-3-*O*-glucoside (Cy3G), cyanidin-3,5-*O*-glucoside (Cy3G5G), peonidin-3-*O*-glucoside (Pn3G), and peonidin-3,5-*O*-glucoside (Pn3G5G) were detected in the blotched parts, while only Cy3G5G and Pn3G5G were detected in the non-blotched parts. This suggests that glucosyltransferases may play a vital role in the four kinds of glucosylated anthocyanins in the blotched parts. Moreover, 2433 differentially expressed genes (DEGs) were obtained from transcriptome analysis of blotched and non-blotched parts, and a key UDP-glycosyltransferase named PhUGT78A22 was identified, which could use Cy3G and Pn3G as substrates to produce Cy3G5G and Pn3G5G, respectively, in vitro. Furthermore, silencing of *PhUGT78A22* reduced the content of anthocyanidin 3,5-*O*-diglucoside in *P*. ‘He Xie’.

**Conclusions:**

A UDP-glycosyltransferase, PhUGT78A22, was identified in *P.* ‘He Xie’, and the molecular mechanism underlying differential distribution of anthocyanins within petals was elucidated. This study provides new insights on the biosynthesis of different kinds of anthocyanins within colorful petals, and helps to explain petal blotch formation, which will facilitate the cultivar breeding with respect to increasing ornamental value. Additionally, it provides a reference for understanding the molecular mechanisms responsible for precise regulation of anthocyanin biosynthesis and distribution patterns.

**Supplementary Information:**

The online version contains supplementary material available at 10.1186/s12870-022-03777-5.

## Background

Flower coloration is one of the most beautiful displays in nature and linked to the evolution and subsequent diversification of flowers by attracting animal pollinators and being involved in biotic and abiotic stress responses, ultimately resulting in the angiosperm diversity [1, 2]. The extraordinary array of colors displayed by flowers mainly results from four types of pigment: chlorophylls, carotenoids, flavonoids, and betalains [3]. The most diverse palette of pigments are flavonoids, particularly anthocyanins, which are widely known to confer the shiny orange, pink, red, violet, and blue colors [4], and their biosynthetic pathways are among the most extensively studied in plants to date. Early in the anthocyanin biosynthetic pathway (ABP), three molecules of malonyl-CoA and one molecule of 4-coumarpyl CoA are condensed by chalcone synthase (CHS) to produce chalcones, which are converted to dihydroflavonols sequentially by chalcone isomerase (CHI), flavanone 3-hydroxylase (F3H), flavonoid 3′-hydroxylase (F3’H), and flavonoid 3′5’-hydroxylase (F3’5’H). In the late stage of ABP, anthocyanidins are synthesized by two enzymes, namely, dihydroflavonol 4-reductase (DFR) and anthocyanin synthase (ANS), then further glycosylated by UDP flavonoid glucosyltransferase (UFGT) and/or methylated by flavonoid *O*-methyltransferases (FOMT). The above ABP enzyme genes are mainly controlled by tissue-specific expression, which are conferred by a MBW protein complex consisting of MYB transcription factors-basic helix-loop-helix (bHLH)-WD40 [2, 5, 6].

*Paeonia*, the only genus in the family Paeoniaceae, is divided into three sections including: *Moutan*, *Onaepia*, and *Paeonia* [7]. Plants in the genus *Paeonia* are worldwide known ornamentals which originated from China and have a long history of cultivation and breeding [8]. In section *Moutan*, two wild species, *P. rockii* and *P. delavayi*, produce flowers with petal blotches, which can also be observed in their offspring [9], as the petal blotches are a dominant genetic trait. Petal blotches occur in various colors and sizes at the base of each petal, which offer a unique ornamental value to its cultivars. We previously compared the anthocyanin composition of blotched and non-blotched parts in 35 cultivars of the section *Moutan*, and found that the most abundant anthocyanins were cyanidin-based glycosides [cyanidin-3-*O*-glucoside (Cy3G) and cyanidin-3,5-*O*-glucoside (Cy3G5G)], however, no anthocyanins were detected in white, non-blotched parts [10]. The transcriptomes of petals with purple blotches and white non-blotched parts of the peony cultivar *P. suffruticosa* ‘Jinrong’ were compared, suggesting that petal blotch formation may be attributed to higher transcriptional levels of *PsCHS*, *PsF3’H*, *PsDFR*, and *PsANS* [11]. In addition, transcriptome analysis of variegated petals of *P. rockii*, *P. ostii*, and their F_1_ hybrids indicated that *CHS*, *DFR,* and *ANS* might be involved in the variegated pigmentation of *Paeonia* flowers [9]. In our previous study, anthocyanin *O*-methyltransferase (AOMT) was identified, and found to be responsible for the methylation of cyanidin glycosides into peonidin glycosides and play an important role in purple coloration of *Paeonia* plants [12]. Despite glycosylated anthocyanins being important for transportation and other important functions, the gene functions of glycosyltransferase have been understudied in *Paeonia*. Moreover, our recent study determined petal blotch color formation is governed by the transcriptional control of *PsCHS* by a MBW complex in the cultivar ‘Qing Hai Hu Yin Bo’ [6]. However, the above studies only focused on colored blotches against white non-blotched parts. Therefore, it is also necessary to study colored petals with colored blotches against a colored (as opposed to white) background to determine how pigment pattern differences are formed within the same petals.

Intersectional hybrids of *Paeonia* offer highly desirable traits for ornamental use. The first intersectional hybrid was created by Toichi Itoh in 1948, opening a new era for cross breeding, and its offspring was named after Itoh to honor his memory. Afterwards, breeders such as Arthur Percy Saunders, considered the father of intersectional hybrids, bred many famous cultivars including ‘First Arrival’, ‘Singing in the Rain’, ‘Scarlet Heaven’, ‘Pastel Splendor’, ‘Hillary’, ‘Canary Brilliants’, and ‘Julia Rose’, among which the famous Itoh hybrids are included. In China, we were lucky to develop an intersectional hybrid named *P.* ‘He Xie’, which resulted from a cross between the section *Moutan* and *Paeonia* [13, 14]. Its phenotype combined the characteristics of the tree peony and the herbaceous peony, and its purple flowers with dark purple blotches provided a higher ornamental commercial value. In our previous study, we identified a ring domain-containing protein (PhRING-H2) in *P.* ‘He Xie’ petals, that physically interacts with PhCHS and is required for PhCHS ubiquitination and degradation, suggesting that post-translational regulation of flavonoid biosynthesis exists in *P.* ‘He Xie’, and provides a theoretical basis for the manipulation of flavonoid biosynthesis in *Paeonia* plants [15]. To further explore the dark purple blotch formation in purple petals, we first compared the types of anthocyanins in blotched and non-blotched parts of petals, then separated the blotched and non-blotched parts of the petals for transcriptome analysis and comparison. Moreover, we found that a key UDP-glycosyltransferase (UGT), PhUGT78A22, can catalyze the transfer of glucose to glucosylated anthocyanins precisely in blotched and non-blotched parts, producing differences in anthocyanin content in the petals of *P.* ‘He Xie’. This study provides new insight on petal blotch patterning against a colorful, non-blotched background, which will illuminate novel color breeding strategies to increase ornamental value, meanwhile, and can be used as a reference for understanding the molecular mechanisms underlying precise control of anthocyanin biosynthesis and accumulation.

## Results

### Different glycosylated anthocyanins were observed in flower petals of *P.* ‘He Xie’ during coloration and opening

**Fig. 1:**
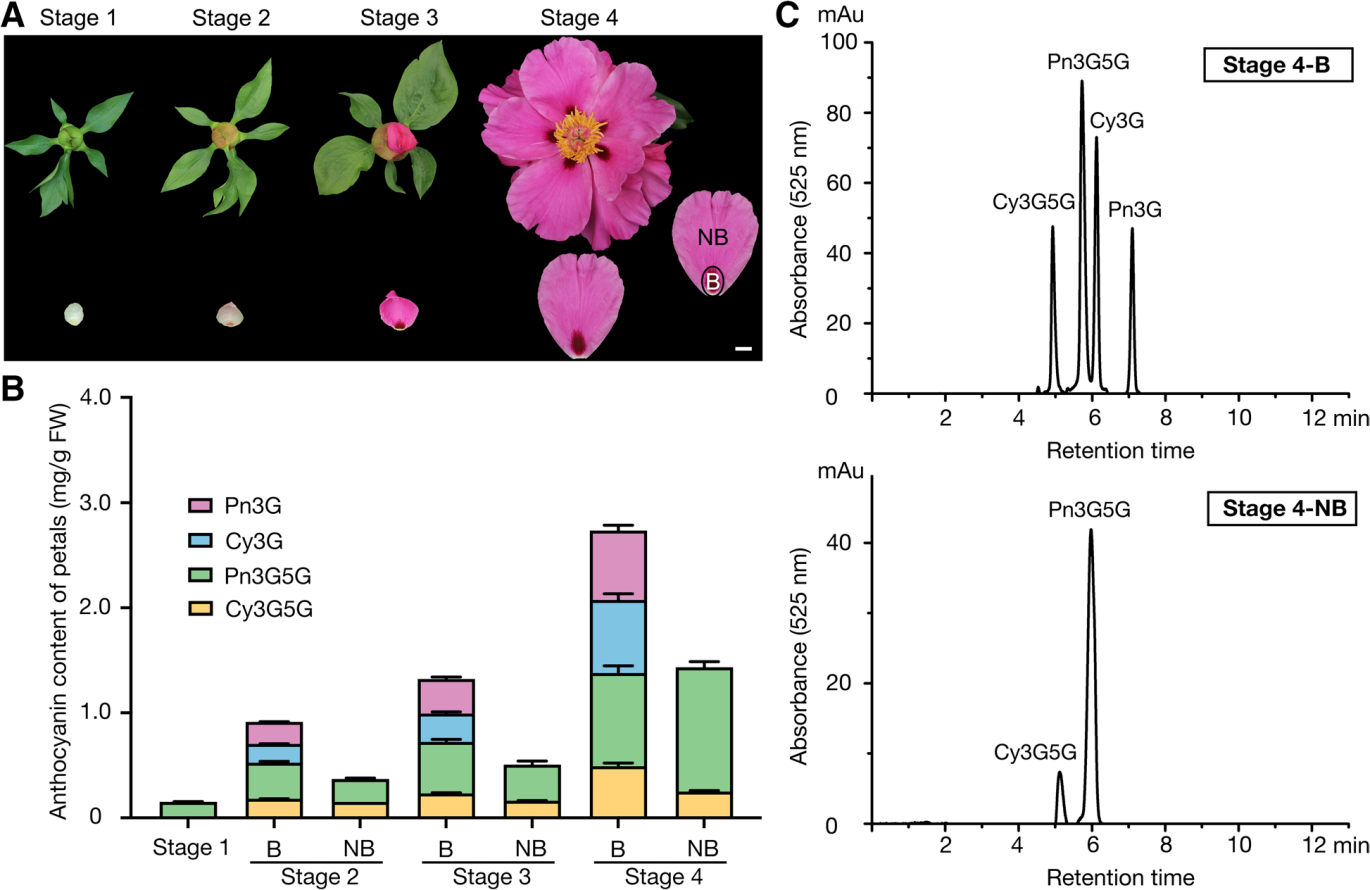
Blotched and non-blotched parts development stages and anthocyanin accumulation patterns in *P.* ‘He Xie’ petals. **A** Phenotypes of different developmental stages of *P.* ‘He Xie’ petals. **B** Anthocyanin accumulation patterns in *P.* ‘He Xie’ petal blotched and non-blotched parts using UPLC-DAD analysis through flower development and opening. Mean values ± SD from three biological replicates (*n* = 3) are shown. **C** Representative UPLC-DAD chromatograms of anthocyanin accumulation in *P.* ‘He Xie’ blotched (upper panels) and non-blotched (lower panels) parts at stage 4. B, blotched part; NB, non-blotched part

In this study, four main developmental stages of *P*. ‘He Xie’ petals were used (Fig. 1A). To investigate pigment accumulation patterns of petals in the blotched and non-blotched parts throughout four developmental stages, the types and concentrations of anthocyanins were measured using ultra-high performance liquid chromatography-diode array detector (UPLC-DAD). We found  that along with petal color develops, the total anthocyanin content increased and reached its highest level at stage 4 (4.17 mg/g FW). From stage 2 to stage 4, the anthocyanin content in the blotched parts was always higher than that in the non-blotched parts, which was 0.91 mg/g FW and 0.37 mg/g FW (stage 2), 1.32 mg/g FW and 0.51 mg/g FW (stage 3), and 2.73 mg/g FW and 1.43 mg/g FW (stage 4) in blotched and non-blotched parts, respectively (Fig. 1B). Four types of anthocyanins were detected in the blotched part, namely Cy3G, Cy3G5G, peonidin-3-*O*-glucoside (Pn3G), and peonidin-3,5-*O*-glucoside (Pn3G5G). However, only two types were detected in the non-blotched part, namely Cy3G5G and Pn3G5G (Fig. 1B & C). These results indicate that anthocyanin glycosylation is different in blotched and non-blotched parts of colored flower petals.

### Petal transcriptome analysis and unigenes identified relating to anthocyanin biosynthesis in *P.* ‘He Xie’

 A total of six samples (three biological replicates for each group) were sequenced and in total, 40.56–45.87 million clean reads were generated with a proper base distribution and mean quality position, and the clean Q30 base rate was higher than 93.44%, indicating good quality sequences for further analysis (Tab. S1). Furthermore, 127,287 unigenes, with an average length of 630 bp after filtering out low quality reads, were obtained and the mapping ratio from the transcriptome was 89.07% using the reference genome of *Vitis vinifera*.

Differentially expressed genes (DEGs) were determined by comparing blotched vs non-blotched parts of *P.* ‘He Xie’ petals, and a total of 2433 unigenes were identified as DEGs, including 1494 up-regulated genes and 939 down-regulated genes. The DEGs were divided into three categories including ‘biological process’, ‘cellular component’, and ‘molecular function’. The most abundant DEGs were annotated as belonging to categories including ‘metabolic process’ (biological process), ‘cell part’ (cellular component), and ‘catalytic activity’ (molecular function) (Fig. S1A). To identify the specific biochemical pathways involved in the pigment accumulation of petals, the DEGs were subjected to Kyoto Encyclopedia of Genes and Genomes (KEGG) pathway for enrichment analysis, which mapped to 285 pathways in the KEGG database. There were seven significantly enriched pathways, including multiple biosynthetic and metabolic pathways, which indicated that there were some differences in biosynthesis and metabolism between blotched and non-blotched parts (Fig. S1B).

**Fig. 2:**
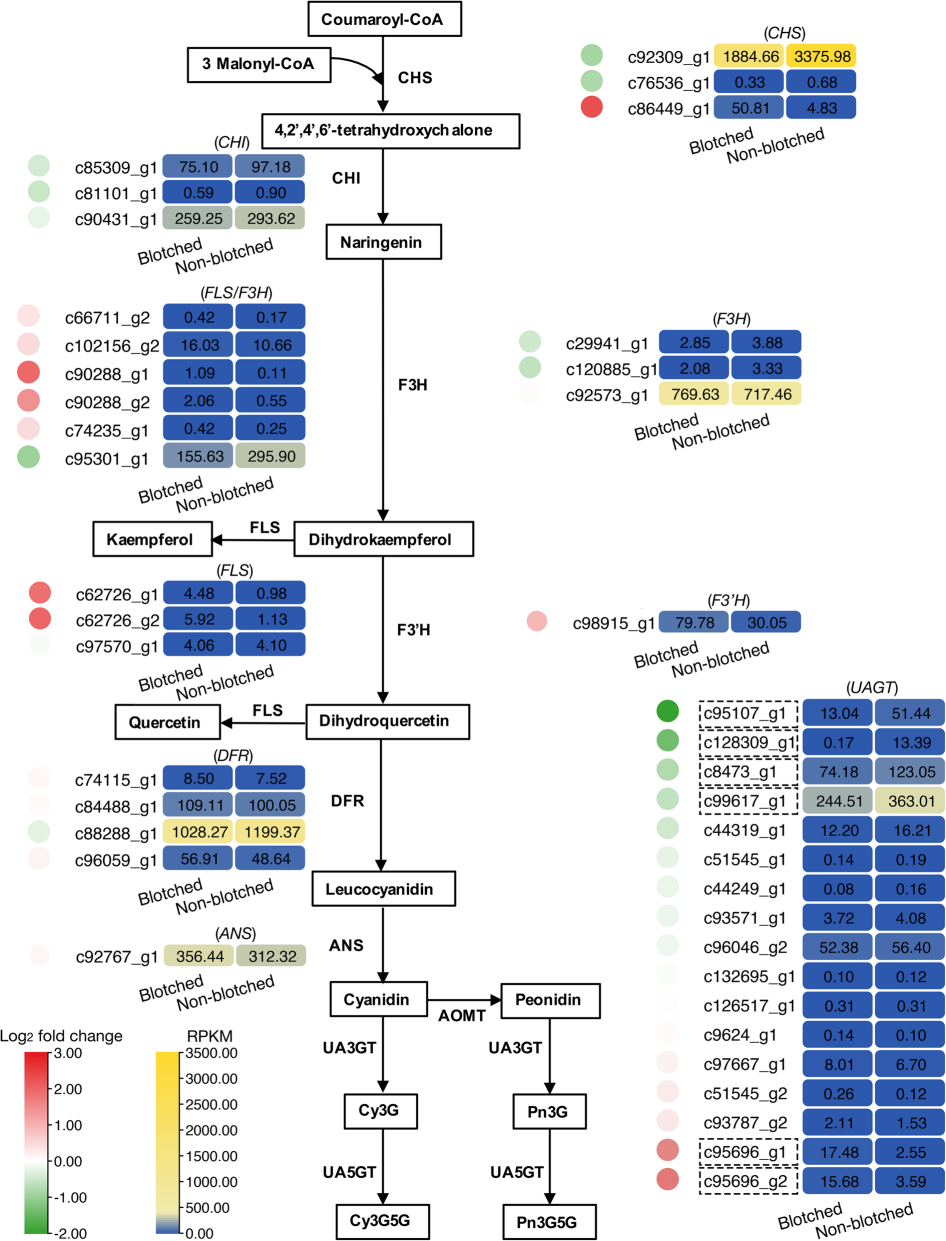
Transcriptome profiles of genes putatively involved in the anthocyanin biosynthetic pathways in blotched and non-blotched parts of *P.* ‘He Xie’ petals. The expression patterns of each gene are shown in the heatmap. The circles on the left of the genes represent the log_2_ fold change value and the round rectangles on the right of the genes represent the RPKM value. The *UAGTs* whose absolute values of the log_2_ fold change are greater than 0.5 are marked with dotted lines

Anthocyanin biosynthesis plays a critical role in determining petal coloration. Therefore, the expression patterns of core genes in the ABP, including *CHS*, *CHI*, *F3H*, *FLS*, *F3’H*, *FLS*, *DFR*, *ANS*, UDP anthocyanin 3-*O*-glucosyltransferase (*UA3GT*), and UDP anthocyanin 5-*O*-glucosyltransferase (*UA5GT*), were studied in detail (Fig. 2; Tab. S2). Among three *CHS* genes, *c92309_g1* and *c76536_g1* were down-regulated, while *c86449_g1* was up-regulated in blotched parts. All three *CHI* genes (*c85309_g1*, *c81101_g1* and *c90431_g1*) were down-regulated in the blotched parts. Among six *FLS*/*F3H* genes, five (*c66711_g2*, *c102156_g2*, *c90288_g1*, *c90288_g2*, and *c74235_g1*) were up-regulated in blotched part, while *c95301_g*1 in the non-blotched part was about two times higher than that in blotched parts. Two *F3H* genes (*c29941_g1* and *c120885_g1*) were down-regulated, *F3’H* (*c98915_g1*) was up-regulated, and two *FLS* genes (*c62726_g1* and *c62726_g2*) were up-regulated in the blotched parts. Among *DFR* and *ANS* genes, there were few differences between blotched and non-blotched parts, except for a slight down-regulation of *c88288_g1* and slight up-regulation of *c96059_g1* in blotched parts, respectively. Seventeen UDP anthocyanin glucosyltransferase (*UAGT*) genes were identified in the transcriptome data. Three *UAGTs* genes (*c132695_g1*, *c126517_g1* and *c9624_g1*) showed little difference of expression levels from transcriptomes between blotched and non-blotched parts, except for 9 *UAGTs*, which were down-regulated (*c95107_g1*, *c128309_g1*, *c8473_g1*, *c99617_g1*, *c44319_g1*, *c51545_g1*, *c44249_g1*, *c93571_g1* and *c96046_g2*) and 5 *UAGTs*, which were up-regulated (*c97667_g1*, *c51545_g2*, *c93787_g2*, *c95696_g1* and *c95696_g2*) in the blotched part, which suggested differential anthocyanin glycosylation occurring between the blotched and non-blotched parts of *P*. ‘He Xie’ petals. Therefore, we focused on glycosyltransferase genes for further examination.

### Expression patterns and sequence analysis of *PhUGTs* involved in anthocyanin biosynthesis in *P*. ‘He Xie’ petals

**Fig. 3:**
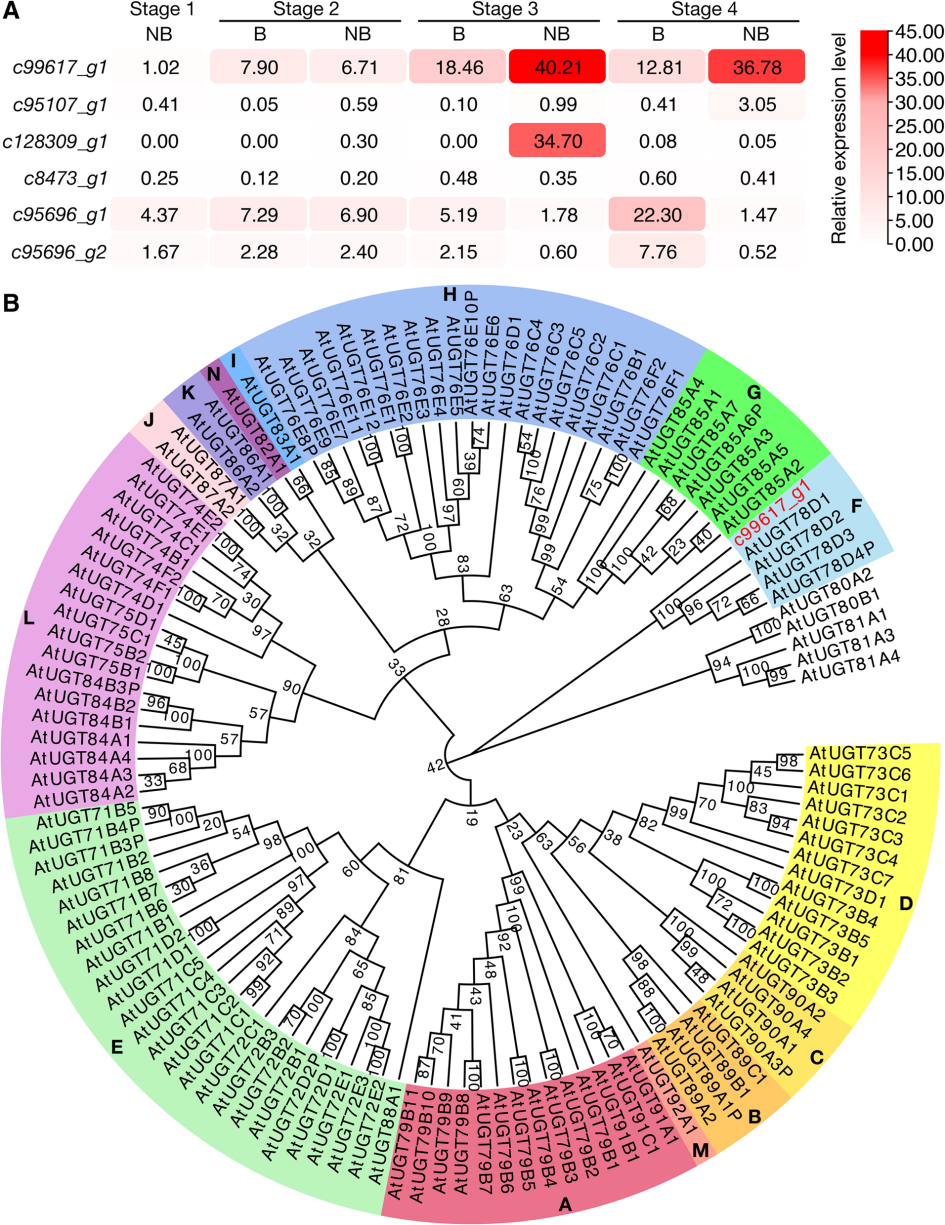
Expression pattern analysis of *UAGT*s and the characterization of PhUGT78A22. **A** RT-qPCR analysis of six *UAGT*s in *P.* ‘He Xie’ blotched and non-blotched parts during flower development. The mean values from three biological replicates (*n* = 3) are shown. **B** A phylogenetic tree of c99617_g1 and UGTs from *A. thaliana*. It was constructed using the Neighbor-Joining method in the MEGA and Evolview. The evolutionary distances were computed using the *p*-distance method and represented as the number of amino acid differences per site. 123 amino acid sequences were used in the analysis and all positions containing gaps and missing data were eliminated

To further study the difference of anthocyanin glycosylation between blotched and non-blotched parts of *P*. ‘He Xie’ petals, expression patterns of *UAGTs* whose absolute values of the log_2_ fold change were greater than 0.5 (*c95107_g1*, *c128309_g1*, *c8473_g1*, *c99617_g1*, *c95696_g1* and *c95696_g2*) were checked using quantitative RT-PCR (RT-qPCR). After blotches appeared at the base of petals, the expression level of *c99617_g1* in non-blotched part was significantly higher than that in blotched parts. The expression level of *c99617_g1* in non-blotched parts at stage 3 was 40.21, which was 1.18-fold greater than that of the blotched part, while, it was 1.87-fold higher in non-blotched versus blotched parts at stage 4. Moreover, unlike other *UAGTs*, *c99617_g1* was highly expressed during *P*. ‘He Xie’ petal coloration. This was consistent with the previous conjecture that anthocyanins with double glucoses were observed in non-blotched parts. The relative expression level of *c8473_g1* was lower than 1.00 during all stages, and *c95107_g1* and *c128309_g1* expression levels were lower than 1.00 during all stages, except for in the non-blotched part during stages 4 (3.05) and 3 (34.07), respectively. The expression levels of *c95696_g1* and *c95696_g2* in blotched parts were higher than in non-blotched parts at stages 3 and 4, which was different from that of *c99617_g1* (Fig. 3A). Considering the anthocyanin types present in non-blotched part and the higher expression levels of *c99617_g1*, it was finally selected for further functional characterization.

Sequence analysis of c99617_g1 showed that its amino acid sequence contained a plant secondary product glycosyltransferase (PSPG) motif (331–374 aa) near the C-terminal domain (Fig. S2). The last glutamine (Q) residue in the PSPG motif, considered to confer specificity for UDP-glucose as the sugar donor, was also observed in c99617_g1 [16]. All of the *Arabidopsis thaliana* UGTs were phylogenetically clustered into 14 groups (labelled A–N) [17, 18]. Phylogenetic analysis showed that c99617_g1 was closest to group F within the *A. thaliana* UGT superfamily, and its closest protein homologs were AtUGT78D1, AtUGT78D2, AtUGT78D3, and AtUGT78D4P (Fig. 3B). We also obtained the name of c99617_g1 from the UGT Nomenclature Committee (https://prime.vetmed.wsu.edu/resources/udp-glucuronsyltransferase-homepage). Combining the evolutionary relationship analysis and UGT Nomenclature Committee naming results, we named c99617_g1 as PhUGT78A22.

**Fig. 4:**
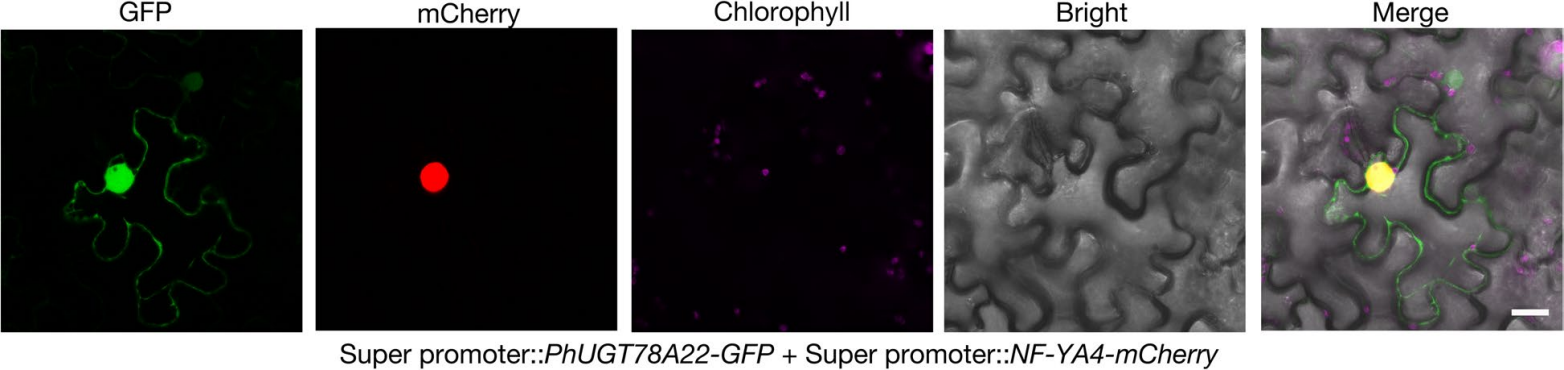
Subcellular localization of PhUGT78A22-GFP heterologously expressed in *Nicotiana benthamiana* leaves. Fluorescence signals were visualized by confocal microscopy 3 d after co-infiltration of the constructs Super promoter:: *PhUGT78A22-GFP* and nuclear marker Super promoter::*NF-YA4-mCherry*. The experiments were performed independently three times, and representative results are shown. Scale bar, 25 μm

To further identify the subcellular localization of PhUGT78A22, PhUGT78A22-GFP was expressed together with a nuclear marker expressed from the construct Super promoter::*NF-YA4-mCherry* and detected a strong GFP signal in the nucleus (Fig. 4).

### Modeling, docking, and substrate specificity analyses of PhUGT78A22

**Fig. 5:**
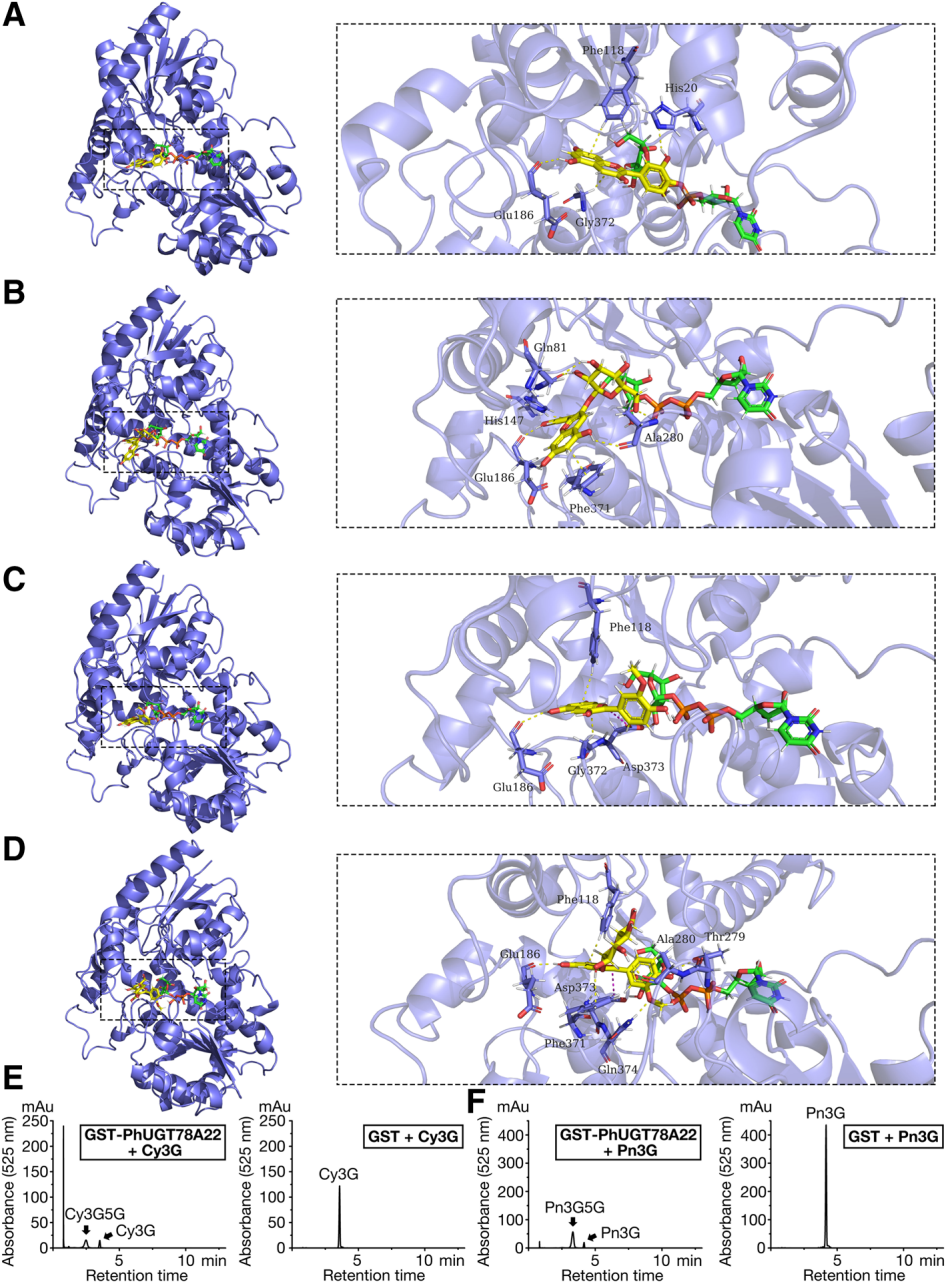
Structural models of PhUGT78A22 docked with UDP-glucose and sugar acceptors and analyses of PhUGT78A22 enzymatic reactions. **A**-**D** Structural models of PhUGT78A22 docked with UDP-glucose and substrate Cy (A), Cy3G (B), Pn (C) and Pn3G (D). The structural model shows the surface binding mode (left) and key amino acid residues for the sugar donor and acceptor positions (right). The substrates are colored yellow and the UDP-glucose is colored green. The surrounding residues in the binding pocket were colored in purple sticks, the backbone of the receptor is depicted as purple cartoon, the hydrogen bonds are depicted as yellow dashed lines, and the salt bridges are depicted as magenta dashed lines. (E & F) Representative UPLC-DAD chromatograms of GST-PhUGT78A22 with Cy3G (E) or Pn3G (F) as substrate (left). Reactions with GST proteins were used as negative controls (right)

The PhUGT78A22 structure was basically consistent with the template UGT structure (PDB code: 2c1x). The root-mean-square deviation (RMSD) value of the 3D structure overlap was 0.111 Å, and both had the same alpha helix and beta strand regions. The identified amino acid sequence of PhUGT78A22 with 2c1x was 66.37% (Fig. S3A & B). The PhUGT78A22 was subjected to the potential interactions analysis between amino acid residues and the UDP-glucose substrate. Nine amino acids of PhUGT78A22 (Thr19, Gly278, Thr279, Ser305, Trp331, Ala332, His349, Glu357, and Asp373) were predicted to interact with the sugar donor UDP-glucose. Specifically, Ser305, Trp331, Ala332, and Glu357 were predicted to putatively interact with uridine groups, while, Thr19, Gly278, and Thr279, His349 putatively interacts with the diphosphate group, and Asp373 interacted with the glucose group of UDP-glucose (Fig. S3C - E, Tab. S3). In total, the docking score between PhUGT78A22 and UDP-glucose was −10.06 kCal/mol (Tab. 1). To examine the role of PhUGT78A22 in the production of anthocyanins in *P*. ‘He Xie’ petals, it was docked with UDP-glucose and substrates of cyanidin (Cy), Cy3G, peonidin (Pn), and Pn3G, respectively. Four amino acids of PhUGT78A22 (His20, Phe118, Glu186, and Gly372) were predicted to interact with the substrate Cy (Fig. 5A, Fig. S4A, Tab. S4), five amino acids (Gln81, His147, Glu186, Ala280, and Phe371) were predicted to interact with the substrate Cy3G (Fig. 5B, Fig. S4B, Tab. S4), four amino acids (Phe118, Glu186, Gly372, and Asp373) were predicted to interact with the substrate Pn (Fig. 5C, Fig. S4C, Tab. S4), and seven amino acids (Phe118, Glu186, Thr279, Ala280, Phe371, Asp373 and Gln374) were predicted to interact with the substrate Pn3G (Fig. 5D, Fig. S4D, Tab. S4). Moreover, the docking scores between PhUGT78A22 + UDP-glucose and Cy, Cy3G, Pn, and Pn3G were −6.66, −8.39, −6.53 and −9.08 kCal/mol, respectively (Tab. 1). The lower the binding energy, the higher the binding affinity obtained will be [19]. The results showed that the binding ability of Cy3G and Pn3G to PhUGT78A22 + UDP-glucose was predicted to be stronger.

**Table 1:** The docking score between PhUGT78A22 and ligands

Receptor	Ligand	Docking score (kCal/mol)
PhUGT78A22	UDP-glucose	−10.06
PhUGT78A22 + UDP-glucose	Cy	− 6.66
PhUGT78A22 + UDP-glucose	Cy3G	− 8.39
PhUGT78A22 + UDP-glucose	Pn	− 6.53
PhUGT78A22 + UDP-glucose	Pn3G	− 9.08

To further analyze the in vitro function of PhUGT78A22, PhUGT78A22 fused with a GST-tag was expressed in *Escherichia coli*, and the isolated protein was evaluated with enzymatic assays. UDP-glucose and two anthocyanins (Cy3G and Pn3G) were tested as sugar donor and substrates, respectively. PhUGT78A22 exhibited catalytic activity toward Cy3G and Pn3G with UDP-glucose as the sugar donor. Analysis of the enzymatic products by UPLC-DAD showed that the recombinant PhUGT78A22 protein catalyzed the conversion of Cy3G into Cy3G5G and Pn3G into Pn3G5G (Fig. 5E & F). The results suggested that PhUGT78A22 catalyzed the transfer of glucose to glucosylated anthocyanins of Cy3G and Pn3G.

### Silencing of *PhUGT78A22* altered Cy3G5G and Pn3G5G biosynthesis in *P.* ‘He Xie’

**Fig. 6:**
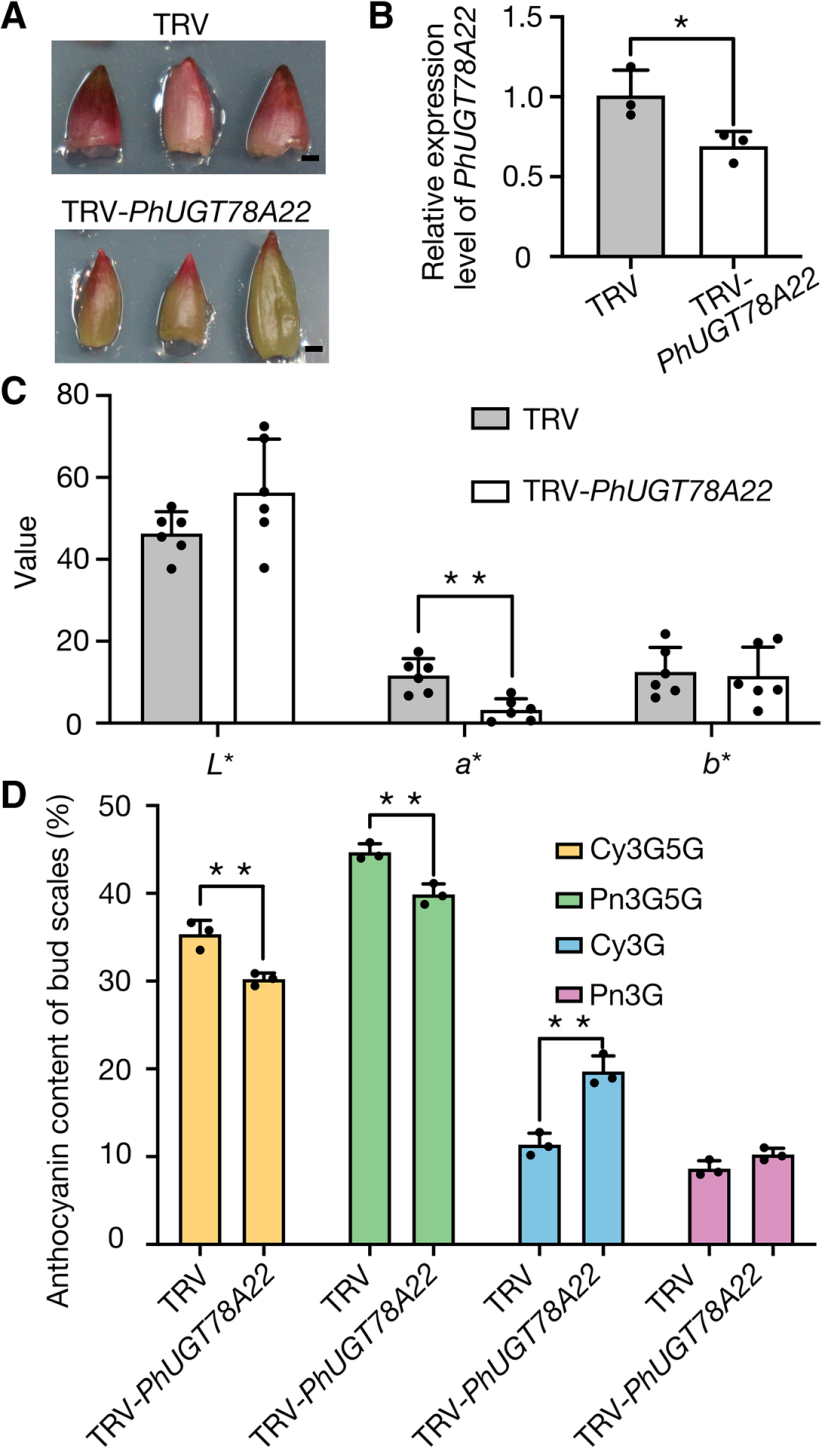
Effects of *PhUGT78A22* silencing on *P.* ‘He Xie’ bud scales. **A** Silencing of *PhUGT78A22* changed bud scale color. Representative images of TRV control and TRV::*PhUGT78A22* treated bud scales were taken 3 d after infiltration. Scale bar, 2 mm. **B** RT-qPCR analysis of *PhUGT78A22* in TRV control and *PhUGT78A22*-silenced *P.* ‘He Xie’ bud scales. One biological sample consisted of a mixture of at least 6 bud scales. The mean values ± SD from three biological replicates (*n* = 3) are shown. Asterisks indicate statistically significant differences (two-sided Student’s *t*-test; *, *P* < 0.05). **C** CIELAB color space analysis of TRV control and *PhUGT78A22*-silenced *P.* ‘He Xie’ bud scales. The mean values ± SD from three biological replicates (*n* = 6) are shown. Asterisks indicate statistically significant differences (two-sided Student’s *t*-test; **, *P* < 0.01). **D** Silencing of *PhUGT78A22* altered anthocyanin accumulation in *P.* ‘He Xie’ bud scales. Anthocyanin accumulation was determined using UPLC-DAD analysis in TRV control and *PhUGT78A22*-silenced bud scales. One biological sample consisted of a mixture of at least 6 bud scales. The mean values ± SD from three biological replicates (n = 3) are shown. Asterisks indicate statistically significant differences (two-sided Student’s *t*-test; **, *P* < 0.01)

To further validate the activity of PhUGT78A22 in vivo, we silenced *PhUGT78A22* using Virus-induced gene silencing (VIGS) technology using a *Tobacco rattle virus* vector in *P*. ‘He Xie’ bud scales. The anthocyanin component of bud scales was the same as that of the blotched part in *P*. ‘He Xie’ petals. Silencing of *PhUGT78A22* changed the bud scale color (Fig. 6A). The expression levels of *PhUGT78A22* was significantly reduced in the TRV::*PhUGT78A22* silenced line, which dropped to 68.51% relative to the TRV control (Fig. 6B). After color measurement, a^*^ value of *PhUGT78A22-*silenced line (3.24) was significantly lower than that of the TRV control (11.65), however, b^*^ and L^*^ values had experienced no significant changes (Fig. 6C), which was consistent with the phenotypic results. To further confirm the changes in anthocyanin content after *PhUGT78A22* silencing, we detected the types and contents of anthocyanins in the TRV::*PhUGT78A22* silenced line. In *PhUGT78A22-*silenced bud scales, Cy3G5G and Pn3G5G were significantly decreased, however, the content of Cy3G was significantly increased compared to the TRV control. Meanwhile, the content of Pn3G was also increased in *PhUGT78A22-*silenced bud scales, although no statistical difference was observed (Fig. 6D). This suggests that the silencing of *PhUGT78A22* will result in the reduction of Cy and Pn bis-glucosidic anthocyanins.

Taken together, our findings suggest a model for how UDP-glycosyltransferase PhUGT78A22 is involved in the transformation of glucose to glucosylated anthocyanins during petal blotch formation in *P*. ‘He Xie’ (Fig. S5). In blotched parts, the expression levels of *PhUGT78A22* are lower, so only a portion of Cy3G and Pn3G can be glycosylated into Cy3G5G and Pn3G5G, respectively. In non-blotched parts, the expression of *PhUGT78A22* is higher, and all Cy3G and Pn3G can be glycosylated into Cy3G5G and Pn3G5G, respectively. The differences in the types and concentrations of glycosylated anthocyanins explains the blotch formation and color differences within *P*. ‘He Xie’ petals.

## Discussion

Floral color blotches have biological significance in attracting insect pollination and driving angiosperm species evolution. In *Paeonia,* variations in color and size of blotches not only increase their ornamental value, but also serves as the basis for species and cultivar classification. Our previous research was focused on the regulatory mechanism of blotch color formations against white non-blotched backgrounds, but little is known about colored petals with colored blotches against a colored (as opposed to white) background, since the anthocyanin types, which were under precise control due to variation in glycosylation between blotched and non-blotched parts. In this study, we aimed at investigating differentially expressed *UGTs* genes and understanding the differences in the types of anthocyanins within the same petals. Additionally, we conducted the functional characterization of *PhUGT78A22* to explain petal blotch formation, which will benefit to breeding with the respect of increasing ornamental value and enrich the understanding of anthocyanin patterning in angiosperms.

The anthocyanin composition of *Paeonia* petals was thoroughly investigated. The flowers of 130 tree peony cultivars (55 red, 28 pink, 38 purple, and 9 white flowered cultivars) from the Zhongyuan cultivation group and 37 (15 red, 10 pink, 8 purple, 3 white, and 1 black flowered cultivars) from Daikon Island were collected in Japan for anthocyanin measurement by Wang et al. [20]. Six anthocyanins, namely Cy3G, Cy3G5G, Pn3G, Pn3G5G, pelargonidin 3-*O*-glucoside (Pg3G), and pelargonidin 3,5-*O*-glucoside (Pg3G5G) constituted the petal pigments and all of the flowers contained peonidin-based glycosides in these tree peony cultivars. Peonidin is the methylated form of cyanidin that produces pink to red pigments [3, 12]. Jia et al. [21] identified five major anthocyanins in 41 herbaceous peony cultivars, namely Cy3G, Cy3G5G, Pn3G, Pn3G5G, and Pg3G5G, which was basically consisted with the results of Wang et al. [20] and Fan et al. [22]. Excluding two cultivars, ‘Huang Jin Lun’ with yellow flowers and ‘Yang Fei Chu Yu’ with white flowers, other 39 cultivars had glucosylated anthocyanins (3,5-*O*-glucoside, 3G5G), among which only 3 cultivars had glucosylated anthocyanidins (3-*O*-glucoside, 3G). Moreover, no 3G-type was detected in 11 cultivars with pink flowers belonging to the “Pn, Cy” group, while, only glucosylated anthocyanins at the 3,5-*O*-position of the backbone (3G5G-type) were obtained [21]. However, in the other “Pn, Cy” group, which included 31 tree peony cultivars from the Xibei (northwest China) cultivation group, excluding 2 cultivars that only contained 3G, all the other cultivars contained the 3G-type and 3G5G-type of Cy and Pn, and the flower colors in this group were pink, purple, white, or black [23]. It can be seen from the above results that the level of glycosylation modification can directly determine the color of *Paeonia* petals. In this study, we identified an intersectional hybrid with only the 3G5G-type of Cy and Pn in non-blotched parts and both the 3G-type and 3G5G-type of Cy and Pn in blotched part, which provided an ideal model for elucidating the role of glycosylation modification in petal color formation in *Paeonia.*

Glycosylation is considered to be the last step in the biosynthetic pathway of the secondary metabolite [24]. The glycosylation process catalyzed by glycosyltransferases, are derived from bacteria, plants, animals and viruses and can be divided into 114 families based on amino acid sequence similarities and catalytic mechanisms in the Carbohydrate-Active Enzymes (CAZymes) Database (Glycosyl Transferase family classification, http://www.cazy.org/GlycosylTransferase-family). Among them, members of the GT family 1 are often referred to as UGTs, which are known to typically transfer a sugar to a diverse array of substrates including hormones, flavonoids, and even pesticides [17, 18, 24]. UGTs have been identified in several higher plants such as peaches, with 16 groups (A - P) of UGTs being found in *Prunus persica* L. Batsch, and two UGTs of group F, namely, Prupe.1G091100 and Prupe.1G091000, were involved in anthocyanin biosynthesis in peach flowers [25]. In this study, we identified PhUGT78A22 belonging to Group F, and to be involved in anthocyanin biosynthesis in *Paeonia.* Some research progress has been made on Group F UGTs, a subfamily with a small number of members, in recent years. In *A. thaliana*, UGT78D1 was identified to catalyze the transfer of rhamnose from UDP-rhamnose to quercetin and kaempferol [26], and UGT78D2 could catalyze the glucosylation of both cyanidins and flavonols as UDP-glucose: flavonoid 3-*O*-glucosyltransferase [27]. In *V. vinifera*, VvGT1 can glycosylate anthocyanidins [28], VvGT5 (UGT78A11) was identified as a UDP-glucuronicacid:flavonol-3-*O*-glucuronosyltransferase and VvGT6 (UGT78A12) as a bifunctional UDP-glucose/UDP-galactose:flavonol-3-*O*-glucosyltransferase/galactosyltransferase [29]. In *Glycine max* (L.) Merr., the UDP-glucose: flavonoid 3-*O*-glucosyltransferase (UGT78K1) only uses anthocyanidins and flavonols as substrates [30]. Here, we firstly identified a group F UGT, PhUGT78A22 in *Paeonia*, and determined its function in glycosylating anthocyanins, which is consistent with the above studies. However, whether it can glycosylate other anthocyanidins or flavonols and participate in processes other than blotch formation in petals remains to be determined. UGT78H2 glycosylated quercetin exclusively using UDP-glucuronic acid and UDP-galactose, but not UDP-glucose [31]. In this study, we determined that PhUGT78A22 transferred glucose to glycosylated anthocyanins with UDP-glucose as the sugar donate, which was different from the results of Chen et al. [31]. This may be due to the differences of species resulting in functional diversification of group F UGTs so as to ensure the accurate modification of corresponding substrates.

Bis-glucosidic anthocyanins are believed to be more hydrophilic and more stable [32]. The present study suggested the high expression level of *PhUGT78A22* would explain all detected anthocyanins being bis-glucosidic ones, which confirmed that most areas of the petal had more hydrophilic and more stable anthocyanins in *P.* ‘He Xie’. In recent years, increasing studies have shown that UGTs play a vital role in plant resistance to stress. Overexpression of *OsUGT90A1* helped to maintain membrane integrity during cold stress, improved freezing survival and tolerance to salt stress, and promoted leaf growth during stress recovery [33]. Ectopic expression of *AtUGT76E1*1 increased flavonoid accumulation and enhanced abiotic stress tolerance to salinity and drought [34]. Ectopic expression of *UFGT2*, first identified from maize, in *A. thaliana* led to increased flavonol contents and enhanced oxidative tolerance [35]. After analyzing comparative genomic and transcriptomic data from three *Brassica* species and *A. thaliana*, a series of UGTs were identified to be involved in plant resistance to cold, drought, and hypoxia stress [36]. Moreover, UGT74E2 was involved in drought and salt stress resistance via ABA and IAA signaling in rice [37]. Chen et al. revealed that UGT75B1 modulated ABA activity by glycosylation in stressful environment [38]. In the present study, we determined the function of PhUGT78A22 in relation to anthocyanin biosynthesis, which may play a similar role in UV-B irradiation stress resistance [25]. Further research is needed to determine whether PhUGT78A22 can be involved in responses to other abiotic stresses, such as drought. In the future, the function of PhUGT78A22 should be fully characterized for its regulatory mechanistic role in flavonoid glycosylation, thus to manipulate flower color variation to improve stress resistance of *Paeonia*, and further synthesize valuable active substances in vitro for health promoting products.

## Conclusions

The present study used an intersectional hybrid named *P.* ‘He Xie’ which has purple flowers with dark purple blotches to explore the mechanism of differential glycosylation of anthocyanin within petals. It was interesting to find that four kinds of anthocyanins (Cy3G, Cy3G5G, Pn3G, and Pn3G5G) in blotched parts, but only Cy3G5G and Pn3G5G in non-blotched part, which suggests glucosyltransferases play a vital role in establishing this difference. Moreover, 2433 DEGs were obtained from transcriptomic analysis of blotched and non-blotched parts, and a key UDP-glycosyltransferase gene named *PhUGT78A22* was identified. It had the conserved PSPG box, suggesting a high affinity to glucosylated anthocyanins at the 3-*O*-postion in the C ring, and used Cy3G and Pn3G as substrates to produce Cy3G5G and Pn3G5G in vitro through molecular docking analysis and enzymatic assays. Furthermore, silencing of *PhUGT78A22* reduced the content of anthocyanidin 3,5-*O*-diglucoside in *P.* ‘He Xie’. This study provides new insights into different types of anthocyanin biosynthesis within same petals, helps explain petal blotch formations and will inspire cultivar breeding with respect of increasing ornamental value. Meanwhile, it provides a reference for understanding the molecular mechanism on precise regulation of anthocyanin biosynthesis and distribution patterns.

## Methods

### Plant materials

In this study, the cultivar *P.* ‘He Xie’ was used, which was grown in Beijing Botanical Garden, Institute of Botany, Chinese Academy of Sciences (Lat. 39°59′ N, 116°12′ E, Alt. 70 m). Flower petals were separated into blotched and non-blotched parts and collected at four developmental stages: 1) the bud is unopened and petals are light yellow-green and lack blotches; 2) the bud is unopened, red blotches beginning to appear at the base of petals, and non-blotched parts are transiting from light yellow green to light pink; 3) the bud is partially opened, blotched parts have deepened in color and expanded to about 0.5 cm in diameter, while the non-blotched parts have turned completely pink; 4) the flower is fully open and blotches have expanded to about 1.0 cm in diameter. Three flowers were collected per stage. Since no blotches are present at stage 1, the whole petal was selected as non-blotched part. Beyond stage 1, blotched and non-blotched parts of every developmental stage were separated into distinct samples. Bud scales were removed from *P.* ‘He Xie’ flower buds referred to Gu et al. [15].

### UPLC-DAD analyses

Tissue samples from the blotched and non-blotched parts of petals at each developmental stage or bud scales were used for anthocyanin analysis as previously described [6] with minor modifications. Specifically, all non-blotched or blotched parts of each flower were collected as a single biological replicate, and the same amount of tissue, by weight, was taken from each biological repetition to quantify anthocyanin concentration. From each sample, 0.2 g of fresh tissue was treated with 1 mL of 0.2% formic acid/methanol (v/v) solution for 20 minutes, ultrasonically homogenized, then incubated in the dark for 2 h. The resulting extract was centrifuged at 12,000 rpm for 5 min and the supernatant was collected. The above steps were repeated until all anthocyanins had been extracted. The supernatant was filtered through a 0.22 μm filter and stored at −20°C. Anthocyanin types and concentrations were determined using an UPLC-DAD (ACQUITY UPLC® I-Class, Waters, Massachusetts, USA). The analytical column was an ACQUITY UPLC® HSS T3 1.8 μm column (Waters, Massachusetts, USA). Four standards were purchased from Solarbio (Beijing, China), namely Cy3G, Cy3G5G, Pn3G, and Pn3G5G. Among them, Cy3G was used as a standard for quantifying anthocyanin concentration through linear regression.

### Transcriptome sequencing and analyses

To identify key genes involved in anthocyanin biosynthesis in the blotched and non-blotched parts, transcriptomic analysis was performed on blotched and non-blotched tissues samples taken from stages 2 to 4 from *P*. ‘He Xie’ petals, using the Illumina sequencing platform. After extracting total RNA, the integrity was evaluated by 1.0% agarose gel electrophoresis, quality checked using a K5500 micro-spectrophotometer (Kaiao, Beijing, China), and the integrity checked again using a 2100 RNA Nano 6000 assay Kit (Agilent Technologies, CA, USA). Library construction and RNA-seq analysis were performed by Annoroad Gene Technology Co. (Beijing, China) using an Illumina platform (CA, USA). Clean reads were obtained by filtering out contaminated and low quality reads, and reads with more than 5% undistinguished bases [39]. Subsequently, De novo transcriptome assembly was performed using Trinity (version 20140717).

The expression levels of unigenes were calculated using the RPKM (Reads Per Kilobase Million Mapped Reads) method. DESeq was used to identify DEGs, and genes with |log_2_Ratio| ≥ 1 and *q* < 0.05 were assigned as differentially expressed. In addition, DEGs were annotated and classified using the Gene Ontology (GO) and KEGG databases.

### RT-qPCR analyses

Total RNA from blotched and non-blotched parts of *P.* ‘He Xie’ petals was extracted using the E.Z.N.A.® Plant RNA Kit (Omega Bio-Tek, GA, USA). cDNA was synthesized using the HiScript II reverse transcriptase kit (Vazyme, Nanjing, China). RT-qPCR reactions were conducted using a StepOne Real-Time PCR System (Applied Biosystems, Carlsbad, USA) in 10 μL reaction mixture containing 2 × M5 HiPer Realtime PCR Super mix (Mei5bio, Beijing, China). *Poβ-Tubulin* (*Poβ-TUB*) was used as an internal control. Each experiment was conducted with three biological repeat. Primers used are listed in Table S5.

### Phylogenetic analyses

Phylogenetic analyses was performed as described previously [17, 18]. All of the *A. thaliana* UGTs were obtained from the CAZymes Database (http://www.cazy.org/Home.html). The phylogenetic tree of c99617_g1 with *A. thaliana* UGTs was constructed using the Neighbor-Joining method in MEGA (version X) and Evolview (http://www.evolgenius.info/evolview.html). In total, 123 amino acid sequences were used in the analysis and all positions containing gaps and missing data were eliminated.

### Subcellular localization of PhUGT78A22

Subcellular localization was performed using Super1300 vector with *Green fluorescent protein* (*GFP*) as the signal protein [40]. The coding region sequence (CDS) of *PhUGT78A22* (1371 bp), with stop codons removed and tagged with GFP, was constructed under a Super promoter [41], then heterologously expressed in *N. benthamiana* leaves through *Agrobacterium tumefaciens* strain GV3101. The constructs Super promoter::*PhUGT78A22-GFP* and nuclear marker Super promoter::*NF-YA4-mCherry* were co-infiltrated using agroinfiltration. GFP and mCherry fluorescence signal were visualized by confocal microscopy (Leica TCS SP5, Germany) 3 days post infiltration. The primer sequences used to make the subcellular localization constructs are listed in Table S5.

### Homology modeling and molecular docking

To determine the molecular basis for the specificity of PhUGT78A22, firstly, the homology model structure of PhUGT78A22 was computed using the SWISS-MODEL server homology modelling pipeline (https://swissmodel.expasy.org/), which uses an anthocyanidin 3-*O*-glucosyltransferase of *V. vinifera* [Protein Data Bank (PDB) code is 2c1x] as a template. The Molecular Operating Environment (MOE) Dock was used for molecular docking of molecules with PhUGT78A22. UDP-glucose was used as the sugar donor. Cy, Cy3G, Pn, and Pn3G were used as the sugar acceptors. The two-dimensional (2D) structures of UDP-glucose, Cy, Cy3G, Pn, and Pn3G were downloaded from PubChem (https://pubchem.ncbi.nlm.nih.gov/) and converted to three-dimensional (3D) structures in MOE through energy minimization, as ligands. The best probable binding mode was visualized by PyMOL (www.pymol.org).

### In vitro enzymatic assays of recombinant PhUGT78A22

In vitro enzymatic assays were performed as described previously [18]. The coding sequence of *PhUGT78A22* was cloned into pGEX4T-2 and expressed in *E. coli* strain Rosetta. Protein expression was induced by adding isopropylthio-*β*-D-galactoside (0.3 mM) to the cell culture, which was then incubated at 120 rpm for 24 h at 16°C. The GST-PhUGT78A22 protein was purified using Glutathione Sepharose 4B (GE Healthcare, PA, USA). UDP-glucose (Solarbio, Beijing, China) was used as the sugar donor. Cy3G and Pn3G (Solarbio, China) were used as the sugar acceptors. The reaction mixture for the PhUGT78A22 enzymatic assay consisted of 100 mM Tris-HCl (pH 7.0), 0.1 mM of each substrate, 2 mM of UDP-glucose, and 3–10 μg of PhUGT78A22 protein, in a 50 μL reaction volume. After incubation for 1 h at 37°C, the reaction was terminated by the addition of 0.2% formic acid/methanol solution (v/v), followed by analysis using a UPLC-DAD assay. The primers used for cloning are listed in Table S5.

### VIGS

VIGS was performed as previously described [15, 42]. A gene-specific fragment of *PhUGT78A22* (327 bp in length) from 3′ end was used to construct the vector TRV2::*PhUGT78A22*. TRV1, TRV2, and TRV2::*PhUGT78A22* were transformed into *A. tumefaciens* strain GV3101, which were then cultured in Luria–Bertani medium (with 50 mg/L rifampicin and 50 mg/L kanamycin) overnight, then harvested by centrifugation at 5000 rpm for 10 min and resuspended in infiltration buffer (10 mM MgCl_2_, 200 mM acetosyringone and 10 mM 2-(N-Morpholino)-ethanesulfonic acid, pH 5.6), and diluted to an A_600_ of 1.5. *A. tumefaciens* cultures containing TRV1 + TRV2::*PhUGT78A22* or TRV1 + TRV2 (as the negative control) were mixed by 1:1, then incubated in the dark at room temperature for 4–6 h before inoculation. *P.* ‘He Xie’ bud scales of the same size and color were collected, submerged in the agrobacterium suspensions, and exposed to a vacuum of −0.9 atm twice, each for 5 min. The infiltrated bud scales were briefly washed with distilled water and placed on solid Murashige and Skoog (MS) medium (41.74 g/L MS, 6.5 g/L agar, pH 5.8; Solarbio, Beijing, China) and cultured in the dark at 8°C for 3 d. Phenotypes were observed, and the expression levels of *PhUGT78A22* and the types and content of anthocyanins in silenced and controlled bud scales was determined following the above procedure. The primers used for VIGS are listed in Table S5.

### Color measurements (CIELAB system)

Chromatic analyses were performed as previously described [12, 43]. The colors were represented as L^*^, a^*^, and b^*^ values. The value of L^*^ is from black (0) to white (100), which represents lightness. The value of a^*^ is from red (positive) to green (negative). The value of b^*^ is from yellow (positive) to blue (negative). Colors were measured using a spectrophotometer NF555 (Nippon Denshoku, Tokyo, Japan).

### Statistical analysis

Statistical analysis was performed in GraphPad Prism (Version 8). All experimental data were tested with a two-sided Student’s *t*-test.

## Supplementary Information

**Additional file 1.**

## Data Availability

The sequence of PhUGT78A22 was enclosed in the Supplementary Information Fig. S2 & S6. Gene sequence data were deposited to National Center for Biotechnology Information with the following accession numbers: *PhUGT78A22* (OM310997, https://www.ncbi.nlm.nih.gov/nuccore/OM310997.1/).

## References

[1] Thomas MM, Rudall PJ, Ellis AG, Savolainen V, Glover BJ (2009). Development of a complex floral trait: the pollinator-attracting petal spots of the beetle daisy, *Gorteria diffusa* (Asteraceae). Am J Bot.

[2] Mekapogu M, Vasamsetti BMK, Kwon O-K, Ahn M-S, Lim S-H, Jung J-A (2020). Anthocyanins in floral colors: biosynthesis and regulation in chrysanthemum flowers. Int J Mol Sci.

[3] Narbona E, del Valle JC, Whittall JB (2021). Painting the green canvas: how pigments produce flower colours. Biochemist.

[4] Castañeda-Ovando A, Ma P-H, de L, Páez-Hernández MaE, Rodríguez JA, Galán-Vidal CA. (2009). Chemical studies of anthocyanins: a review. Food Chem.

[5] Grotewold E (2006). The genetics and biochemistry of floral pigments. Annu Rev Plant Biol.

[6] Gu Z, Zhu J, Hao Q, Yuan Y-W, Duan Y-W, Men S (2019). A novel R2R3-MYB transcription factor contributes to petal blotch formation by regulating organ-specific expression of *PsCHS* in tree peony ( *Paeonia suffruticosa* ). Plant Cell Physiol.

[7] Hao Q, Liu Z-A, Shu Q-Y, Zhang R, De Rick J, Wang L-S (2008). Studies on *Paeonia* cultivars and hybrids identification based on SRAP analysis. Hereditas..

[8] Zhou S-L, Zou X-H, Zhou Z-Q, Liu J, Xu C, Yu J (2014). Multiple species of wild tree peonies gave rise to the ‘king of flowers’, *Paeonia suffruticosa* Andrews. Proc R Soc B.

[9] Shi Q, Li L, Zhang X, Luo J, Li X, Zhai L (2017). Biochemical and comparative transcriptomic analyses identify candidate genes related to variegation formation in *Paeonia rockii*. Molecules..

[10] Zhang J, Wang L, Shu Q, Liu Z, Li C, Zhang J (2007). Comparison of anthocyanins in non-blotches and blotches of the petals of Xibei tree peony. Sci Hortic.

[11] Zhang Y, Cheng Y, Ya H, Xu S, Han J. Transcriptome sequencing of purple petal spot region in tree peony reveals differentially expressed anthocyanin structural genes. Front. Plant Sci. 2015;6. 10.3389/fpls.2015.00964 PMC4631938 26583029

[12] Du H, Wu J, Ji K-X, Zeng Q-Y, Bhuiya M-W, Su S (2015). Methylation mediated by an anthocyanin, *O*-methyltransferase, is involved in purple flower coloration in *Paeonia*. J Exp Bot.

[13] Hao Q, Liu Z, Shu Q-Y, Wang L-S, Chen F (2008). Identification of intersectional hybrid between section *Moutan* and section *Paeonia* found in China for the first time. Acta Horticulturae Sinica.

[14] Tong N, Peng L, Liu Z, Li Y, Zhou X, Wang X (2021). Comparative transcriptomic analysis of genes involved in stem lignin biosynthesis in woody and herbaceous *Paeonia* species. Physiol Plant.

[15] Gu Z, Men S, Zhu J, Hao Q, Tong N, Liu Z-A (2019). Chalcone synthase is ubiquitinated and degraded via interactions with a RING-H2 protein in petals of *Paeonia* ‘He Xie’. J Exp Bot..

[16] Kubo A, Arai Y, Nagashima S, Yoshikawa T (2004). Alteration of sugar donor specificities of plant glycosyltransferases by a single point mutation. Arch Biochem Biophys.

[17] Ross J, Li Y, Lim E-K, Bowles DJ. Higher plant glycosyltransferases. Genome Biol. 2001;2:3004.1–6. 10.1186/gb-2001-2-2-reviews3004 PMC138907 11182895

[18] Yin Q, Shen G, Chang Z, Tang Y, Gao H, Pang Y (2017). Involvement of three putative glucosyltransferases from the UGT72 family in flavonol glucoside/rhamnoside biosynthesis in *Lotus japonicus* seeds. J Exp Bot.

[19] Guleria P, Yadav SK (2013). Agrobacterium mediated transient gene silencing (AMTS) in *Stevia rebaudiana*: insights into steviol glycoside biosynthesis pathway. PLoS One.

[20] Wang L-S, Shiraishi A, Hashimoto F, Aoki N, Shimizu K, Sakata Y (2001). Analysis of petal anthocyanins to investigate flower coloration of Zhongyuan (Chinese) and Daikon Island (Japanese) tree peony cultivars. J Plant Res.

[21] Jia N, Shu Q-Y, Wang L-S, Du H, Xu Y-J, Liu Z-A (2008). Analysis of petal anthocyanins to investigate coloration mechanism in herbaceous peony cultivars. Sci Hortic.

[22] Fan J, Zhu W, Kang H, Ma H, Tao G (2012). Flavonoid constituents and antioxidant capacity in flowers of different Zhongyuan tree penoy cultivars. J Funct Foods.

[23] Wang L-S, Hashimoto F, Shiraishi A, Aoki N (2004). Chemical taxonomy of the Xibei tree peony from China by floral pigmentation. J Plant Res.

[24] Khorolragchaa A, Kim Y-J, Rahimi S, Sukweenadhi J, Jang M-G, Yang D-C (2014). Grouping and characterization of putative glycosyltransferase genes from *Panax ginseng* Meyer. Gene..

[25] Wu B, Gao L, Gao J, Xu Y, Liu H, Cao X, et al. Genome-wide identification, expression patterns, and functional analysis of UDP glycosyltransferase family in peach (*Prunus persica* L. Batsch). Front. Plant Sci. 2017;8. 10.3389/fpls.2017.00389 PMC5360731 28382047

[26] Jones P, Messner B, Nakajima J-I, Schäffner AR, Saito K (2003). UGT73C6 and UGT78D1, glycosyltransferases involved in flavonol glycoside biosynthesis in *Arabidopsis thaliana*. J Biol Chem.

[27] Tohge T, Nishiyama Y, Hirai MY, Yano M, Nakajima J, Awazuhara M (2005). Functional genomics by integrated analysis of metabolome and transcriptome of Arabidopsis plants over-expressing an MYB transcription factor: metabolomics and transcriptomics. Plant J.

[28] Offen W, Martinez-Fleites C, Yang M, Kiat-Lim E, Davis BG, Tarling CA (2006). Structure of a flavonoid glucosyltransferase reveals the basis for plant natural product modification. EMBO J.

[29] Ono E, Homma Y, Horikawa M, Kunikane-Doi S, Imai H, Takahashi S (2010). Functional differentiation of the glycosyltransferases that contribute to the chemical diversity of bioactive flavonol glycosides in grapevines (*Vitis vinifera*). Plant Cell.

[30] Kovinich N, Saleem A, Arnason JT, Miki B (2010). Functional characterization of a UDP-glucose:flavonoid 3-*O*-glucosyltransferase from the seed coat of black soybean (*Glycine max* (L.) Merr.). Phytochemistry..

[31] Chen Q, Liu X, Hu Y, Wang Y, Sun B, Chen T (2021). Broaden the sugar donor selectivity of blackberry glycosyltransferase UGT78H2 through residual substitutions. Int J Biol Macromol.

[32] He F, Chen W-K, Yu K-J, Ji X-N, Duan C-Q, Reeves MJ (2015). Molecular and biochemical characterization of the UDP-glucose: anthocyanin 5-*O*-glucosyltransferase from *Vitis amurensis*. Phytochemistry..

[33] Shi Y, Phan H, Liu Y, Cao S, Zhang Z, Chu C (2020). Glycosyltransferase *OsUGT90A1* helps protect the plasma membrane during chilling stress in rice. J Exp Bot.

[34] Li Q, Yu H-M, Meng X-F, Lin J-S, Li Y-J, Hou B-K (2018). Ectopic expression of glycosyltransferase UGT76E11 increases flavonoid accumulation and enhances abiotic stress tolerance in Arabidopsis. Plant Biol.

[35] Li Y, Li P, Wang T, Zhang F, Huang X, Hou B (2018). The maize secondary metabolism glycosyltransferase UFGT2 modifies flavonols and contributes to plant acclimation to abiotic stresses. Ann Bot.

[36] Rehman HM, Nawaz MA, Shah ZH, Ludwig-Müller J, Chung G, Ahmad MQ (2018). Comparative genomic and transcriptomic analyses of Family-1 UDP glycosyltransferase in three *Brassica* species and *Arabidopsis* indicates stress-responsive regulation. Sci Rep.

[37] Wang T, Li P, Mu T, Dong G, Zheng C, Jin S (2020). Overexpression of *UGT74E2*, an Arabidopsis IBA glycosyltransferase, enhances seed germination and modulates stress tolerance via ABA signaling in rice. Int J Mol Sci.

[38] Chen T-T, Liu F-F, Xiao D-W, Jiang X-Y, Li P, Zhao S-M (2020). The Arabidopsis UDP-glycosyltransferase75B1, conjugates abscisic acid and affects plant response to abiotic stresses. Plant Mol Biol.

[39] Brown J, Pirrung M, McCue LA (2017). FQC dashboard: integrates FastQC results into a web-based, interactive, and extensible FASTQ quality control tool. Bioinformatics..

[40] Li Y, Wang X, Zhang X, Liu Z, Peng L, Hao Q (2022). ABSCISIC ACID-INSENSITIVE 5-*ω3 FATTY ACID DESATURASE3* module regulates unsaturated fatty acids biosynthesis in *Paeonia ostii*. Plant Sci.

[41] Lee L-Y, Kononov ME, Bassuner B, Frame BR, Wane K, Gelvin SB (2007). Novel plant transformation vectors containing the superpromoter. Plant Physiol.

[42] Chen J, Li Y, Li Y, Li Y, Wang Y, Jiang C (2021). AUXIN RESPONSE FACTOR 18–HISTONE DEACETYLASE 6 module regulates floral organ identity in rose (*Rosa hybrida*). Plant Physiol..

[43] Gonnet J-F (1998). Colour effects of co-pigmentation of anthocyanins revisited—1. A colorimetric definition using the CIELAB scale. Food Chem.

