# Supplementary material for: PhUGT78A22, a novel glycosyltransferase in Paeonia ‘He Xie’, can catalyze the transfer of glucose to glucosylated anthocyanins during petal blotch formation
Source: BMC Plant Biol. 2022 Aug 18;22:405. doi: 10.1186/s12870-022-03777-5 (PMC9386992; doi:10.1186/s12870-022-03777-5)
Supplement: Supplementary file 1 — Additional file 1. [file 12870_2022_3777_MOESM1_ESM.pdf]

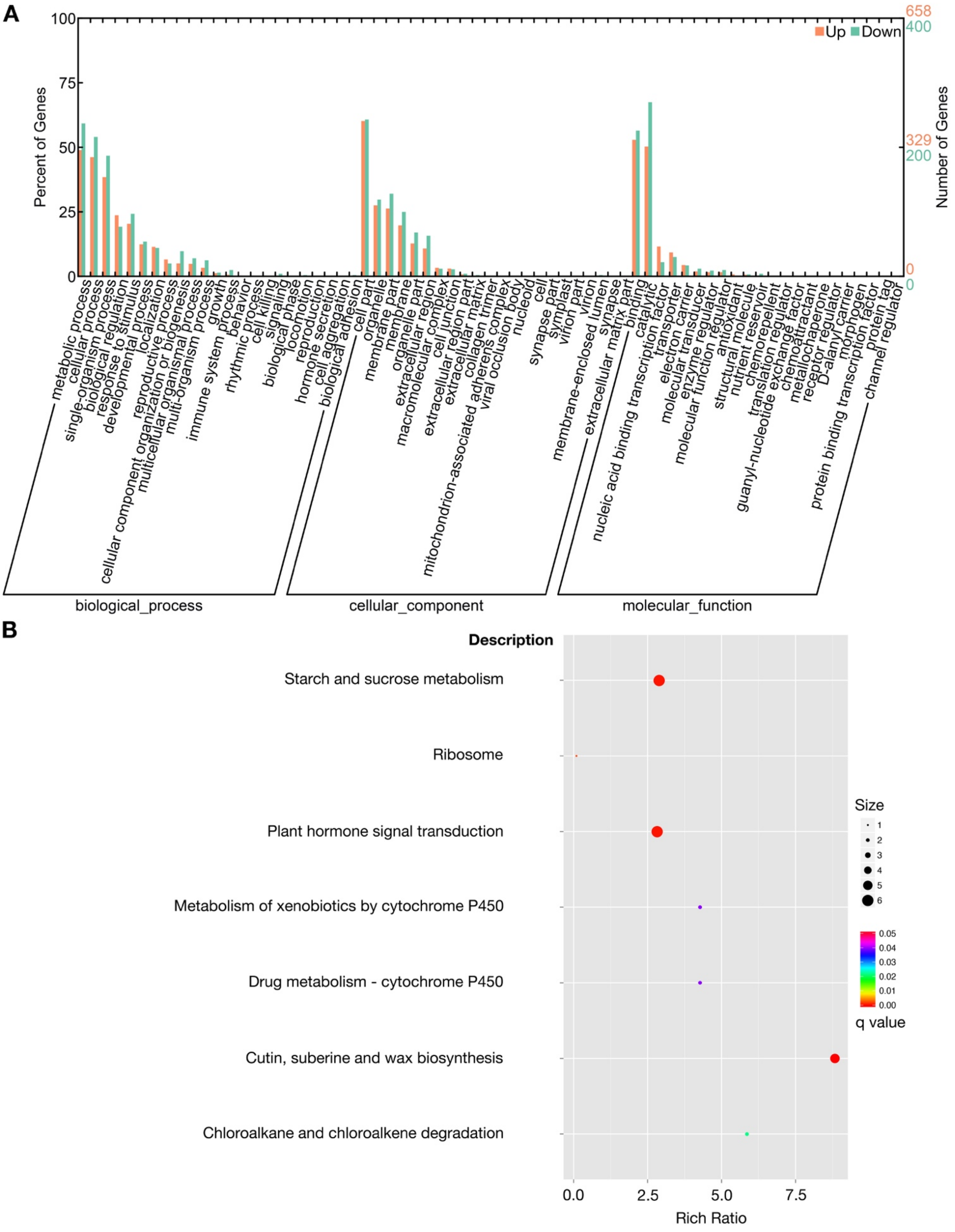


**Fig. S1 GO and KEGG enrichment analysis of DEGs from transcriptome between blotched and non-blotched parts of *P.* 'He Xie' petals.**

Genes were analyzed by DEseq2, and genes whose $|\log_2 \text{Ratio}| \geq 1$ and $q < 0.05$ were chosen as DEGs. DEGs were analyzed by GO (A) and KEGG (B) enrichment analysis, respectively.

MTSMTNEPHVAVFAFPFGTHAAPLLTIIRYLSKAAPNVHFSFFSTAESNTMIFSN
SNNDAVKAYNVSDGVPDKYVFTGKHQEKIDLFMKAAPENFRKCMEAAVAET
GRKVSCLVTDGFFWFAAEMAEEMGVPWVPFWTAGPNSLSTHVLTDFIRDKV
GAGGIEGREEEPLAFIPGMSKLRLRDLPEGILVGNLNSIFSTMLHKMGQMLPQ
ATAVFINSFEELDPTLTNDLNSKFKKFLNIGPFNLLSPLPPPSTPDANNCLSWLN
DQKAESVAYISFGTAATPPPTEILAIAEALEASGVAFLWSLKDHLNVHLPKGFL
DKTRACGMVVPWAPQLQILAHGAVGVFVTHCGWNSVLESIGGGVPMICRPFF
GDQKLNACMVEDVWEIGVKIDGGVFTKNGLISSLDLVLSQEKGKKMRGEIRG
LKGLAEKAVGPQGSSTENLKTLLSLVSRHKDIA

**Fig. S2 Amino acid sequence and the PSPG motifs of c99617_g1.**

The underlined nucleotides represent the putative C-terminal UDP-binding motif for PSPG.

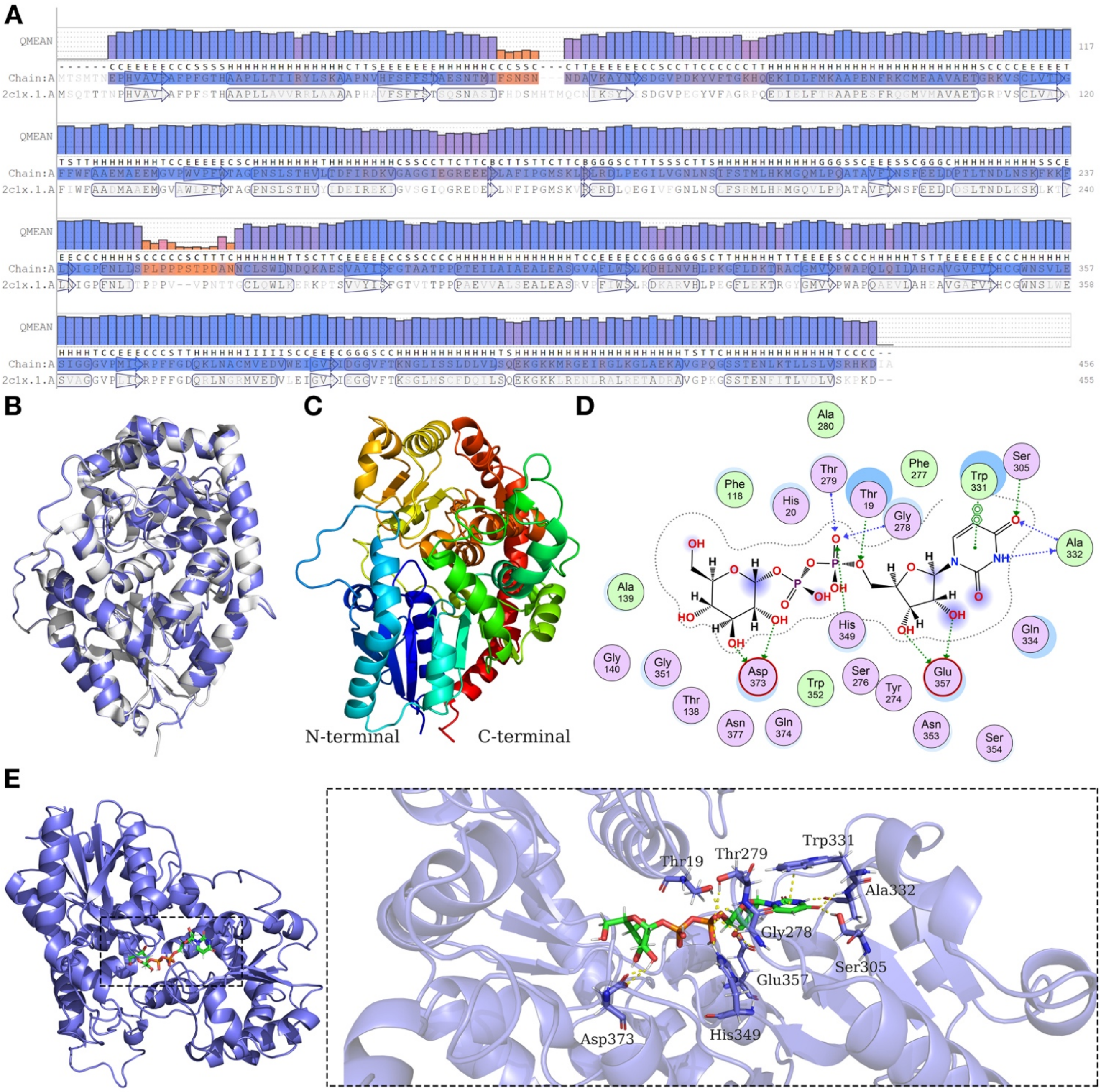


**Fig. S3 Structural analysis of PhUGT78A22 modeling results and the binding mode of UDP-glucose and PhUGT78A22.**

(A) The structure-based sequence comparison between PhUGT78A22 and the template (PDB code: 2c1x). The same or similar residues were highlighted in blue and dissimilar ones were highlighted in orange, the darker color indicated more similar or dissimilar residues. The sequence corresponding to alpha-helix regions were marked by gray line. (B) The result of superposition of the PhUGT78A22 model structure and 2c1x. The PhUGT78A22 structure was shown in blue, the 2c1x was shown in white. (C) The

homology model marked the N-terminal and C-terminal of PhUGT78A22. (D) The 2D structural model of PhUGT78A22 docked with UDP-glucose. The gray dashed lines represented proximity contour, the blue dashed lines represented the backbone atom formed a hydrogen bond with ligand, and the green dashed lines represented the sidechain atom formed a hydrogen bond with ligand. (E) The 3D structural model of PhUGT78A22 docked with UDP-glucose. The structural model showed the surface binding mode (left) and its details (right). The UDP-glucose was colored in green. The surrounding residues in the binding pocket were colored in purple sticks, the backbone of the receptor was depicted as purple cartoon, and the hydrogen bonds were depicted as yellow dashed lines.

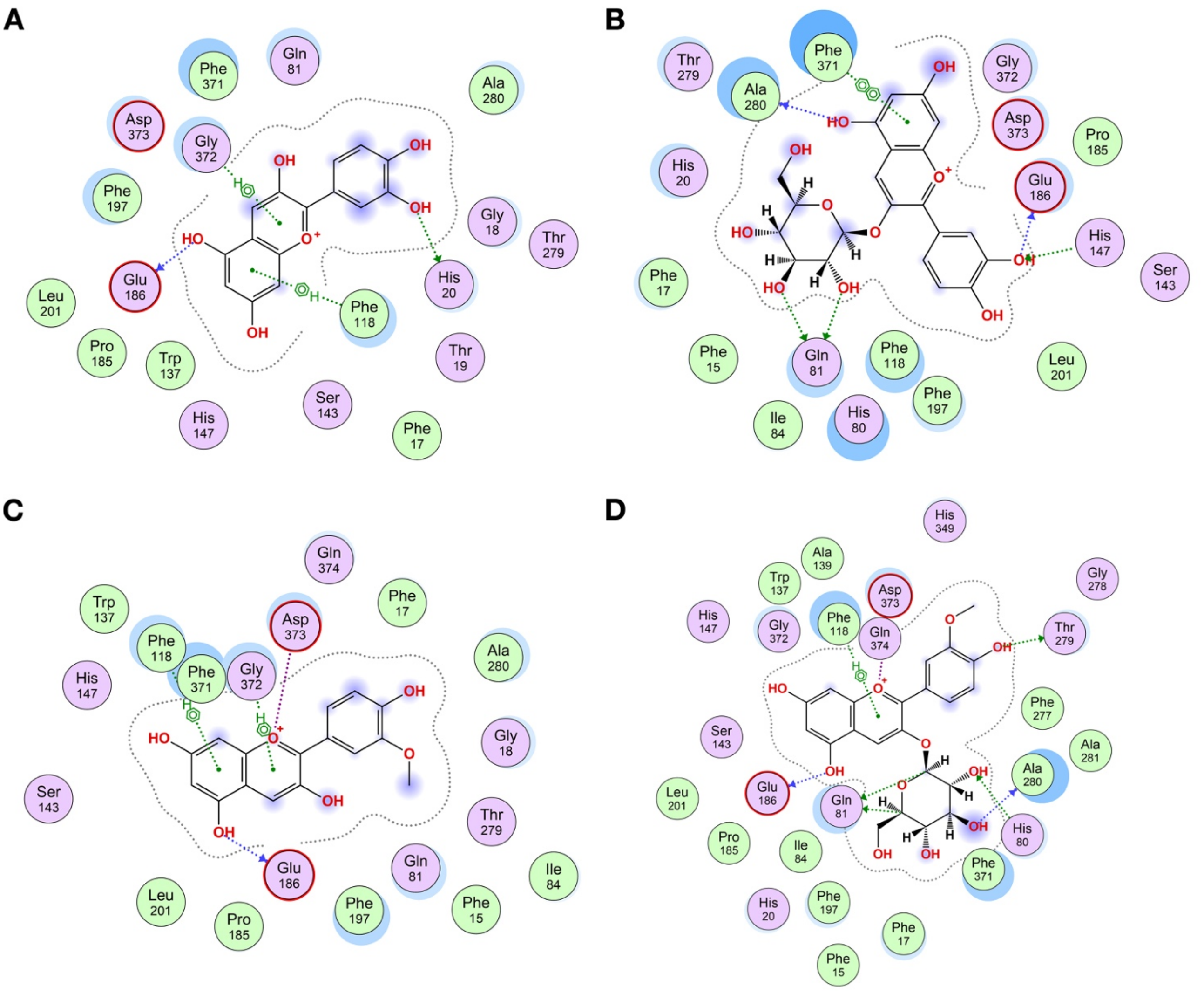


**Fig. S4 The 2D structural model of PhUGT78A22 docked with UDP-glucose and sugar acceptors.**

The 2D binding mode of sugar acceptors Cy (A) , Cy3G (B) , Pn (C) and Pn3G (D) with PhUGT78A22 + UDP-glucose, respectively. The gray dashed lines represented proximity contour, the blue dashed lines represented the backbone atom formed a hydrogen bond with ligand, and the green dashed lines represented the sidechain atom formed a hydrogen bond with ligand.

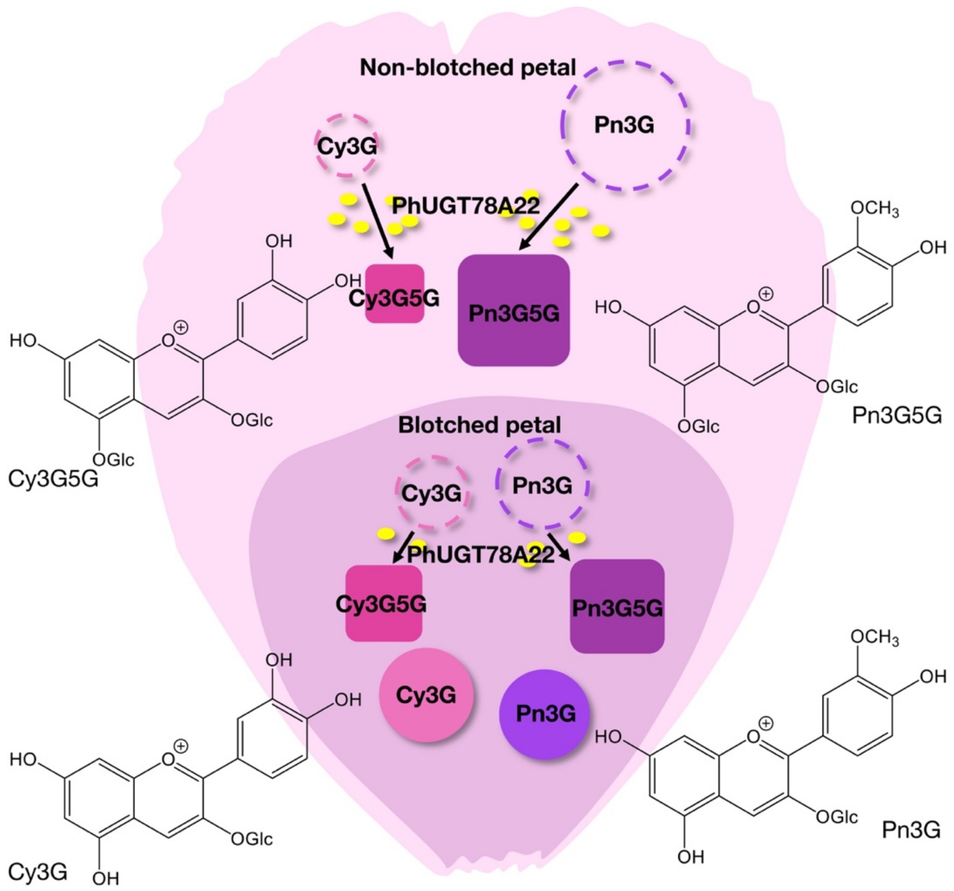


**Fig. S5 Proposed model of PhUGT78A22 in *P*. 'He Xie' petal blotch formation.**

A glycosyltransferase PhUGT78A22 can catalyze the transfer of glucose to glucosylated anthocyanins in *P.* 'He Xie' during petal blotch formation. Blotched part has high accumulation of Cy3G and Pn3G, while has low expression level of *PhUGT78A22*, which results in only part of Cy3G and Pn3G can be glycosylated to Cy3G5G and Pn3G5G. Non-blotched part has low accumulation of Cy3G and Pn3G, while has high expression level of *PhUGT78A22*, which results in all of Cy3G and Pn3G can be glycosylated to Cy3G5G and Pn3G5G. The size of the circles or rectangles represents anthocyanin relative concentration in the tissue.

ATGACGTCGATGACTAACGAGCCACACGTGGCGGTCTTCGCCTTCCCCTTCGGCACAC
ATGCCGCTCCCCTCCTTACCATCATCCGCTACCTTTCGAAAGCCGCCCCAAACGTTCAC
TTCTCATTCTTTAGCACCGCCGAATCCAATACCATGATATTCTCCAACTCCAACAACGAC
GCCGTAAAGGCCTATAATGTTTCTGATGGCGTGCCGGATAAGTACGTGTTCACCGGGAA
GCACCAGGAGAAAATCGACCTTTTCATGAAAGCGGCGCCGGAGAATTTTCGGAAGTG
TATGGAGGCGGCGGTGGCGGAGACGGGAAGGAAGGTGAGCTGTTTAGTGACGGATGG
ATTCTTTTGGTTTGCGGCAGAGATGGCTGAGGAGATGGGGGTGCCTTGGGTGCCGTTT
TGGACTGCTGGGCCTAACTCACTTTCCACTCATGTCCTCACTGATTTCATCCGAGACAA
GGTTGGAGCTGGAGGTATTGAAGGGAGAGAAGAAGAACCACTTGCATTCATTCCAGG
AATGTCCAAATTACGCCTCCGGGACTTGCCGGAAGGAATCCTCGTCGGAAACTTGAAC
TCCATCTTCTCAACCATGCTACACAAAATGGGACAAATGCTGCCACAAGCAACCGCGG
TTTTTATCAACTCCTTCGAAGAATTAGATCCTACTTTAACAAATGACTTAAACTCCAAGT
TCAAAAAATTCCTAAACATTGGCCCTTTTAATCTATTATCACCGTTACCGCCACCATCTA
CGCCTGATGCAAATAATTGCCTGTCATGGCTCAATGACCAAAAGGCTGAATCCGTCGC
GTACATCAGCTTTGGCACGGCTGCGACTCCACCTCCAACCGAGATTTTAGCTATAGCAG
AAGCATTAGAAGCAAGTGGGGTTGCCTTTTTATGGTCACTTAAGGACCATTTAAATGTG
CATTTACCAAAAGGGTTTTTAGACAAAACAAGAGCATGTGGAATGGTTGTGCCATGGG
CTCCTCAATTACAAATACTAGCACATGGTGCTGTTGGGGTGTTTGTAACGCATTGTGGT
TGGAACTCTGTACTAGAGAGTATCGGAGGTGGAGTGCCTATGATTTGTAGGCCATTCTT
CGGCGATCAAAAGTTGAACGCGTGTATGGTGGAGGACGTGTGGGAAATTGGTGTGAA
AATCGATGGTGGAGTATTTACGAAGAATGGGTTAATAAGTAGTTTGGATCTAGTTTTATC
ACAAGAAAAGGGAAAGAAGATGAGGGGGGAGATCAGAGGGTTGAAAGGACTTGCTG
AAAAAGCGGTCGGACCGCAAGGGAGTTCTACTGAGAATTTGAAAACTTTGTTGAGTC
TAGTATCAAGGCATAAGGATATTGCTTAA

**Fig. S6 CDS of *PhUGT78A22*.**

**Tab. S1 The basic data of transcriptome of blotched and non-blotched parts of *P.* 'He Xie' petals.**

| #Samples | Blotched_1 | Blotched_2 | Blotched_3 | Non-blotched_1 | Non-blotched_2 | Non-blotched_3 |
|---|---|---|---|---|---|---|
| Raw Reads Number | 65720662 | 67523174 | 60348980 | 60971366 | 66078410 | 66161608 |
| Raw Bases Number | 9858099300 | 10128476100 | 9052347000 | 9145704900 | 9911761500 | 9924241200 |
| Raw Reads Length (bp) | 150 | 150 | 150 | 150 | 150 | 150 |
| Clean Reads Number | 45118770 | 42502152 | 40561764 | 45814740 | 44838922 | 45869002 |
| Clean Bases Number | 6767815500 | 6375322800 | 6084264600 | 6872211000 | 6725838300 | 6880350300 |
| Clean Reads Length (bp) | 150 | 150 | 150 | 150 | 150 | 150 |
| Clean Reads Rate (%) | 68.65 | 62.94 | 67.21 | 75.14 | 67.86 | 69.33 |
| Adapter Polluted Reads Number | 7283992 | 12557962 | 8181350 | 1970986 | 7926536 | 6339964 |
| Adapter Polluted Reads Rate (%) | 11.08 | 18.60 | 13.56 | 3.23 | 12.00 | 9.58 |
| Ns Reads Number | 256628 | 228680 | 215374 | 240168 | 239712 | 256928 |
| Ns Reads Rate (%) | 0.39 | 0.34 | 0.36 | 0.39 | 0.36 | 0.39 |
| Low-quality Reads Number | 13061270 | 12234380 | 11390492 | 12945470 | 13073238 | 13695712 |
| Low-quality Reads Rate (%) | 19.87 | 18.12 | 18.87 | 21.23 | 19.78 | 20.70 |
| Raw Q30 Bases Rate (%) | 87.38 | 87.68 | 87.77 | 87.57 | 87.18 | 87.28 |
| Clean Q30 Bases Rate (%) | 93.44 | 93.51 | 93.59 | 93.6 | 93.44 | 93.51 |

**Tab. S2 The expression levels of genes related to anthocyanin biosynthetic pathway of blotched and non-blotched parts of *P.* 'He Xie' petals.**

| Gene | B RPKM mean | NB RPKM mean | $log_2$ Fold Change | Description |
|---|---|---|---|---|
| | | *CHS* | | |
| *c92309_g1* | 1884.66 | 3375.98 | -0.88 | chalcone synthase (CHS) mRNA, complete cds [*Paeonia lactiflora*] |
| *c76536_g1* | 0.33 | 0.68 | -0.80 | PREDICTED: chalcone synthase (LOC100259793), transcript variant X2, mRNA [*Vitis vinifera*] |
| *c86449_g1* | 50.81 | 4.83 | 2.30 | chalcone synthase (CHS) mRNA, complete cds [*Camellia sinensis*] |
| | | *CHI* | | |
| *c85309_g1* | 75.10 | 97.18 | -0.41 | chalcone isomerase mRNA, complete cds [*Paeonia suffruticosa*] |
| *c81101_g1* | 0.59 | 0.90 | -0.52 | chalcone isomerase [*Paeonia suffruticosa*] |
| *c90431_g1* | 259.25 | 293.62 | -0.22 | chalcone isomerase [*Gossypium hirsutum*] |
| | | *F3H* | | |
| *c29941_g1* | 2.85 | 3.88 | -0.48 | flavanone 3-hydroxylase [*Paeonia suffruticosa*] |
| *c120885_g1* | 2.08 | 3.33 | -0.60 | flavanone 3-hydroxylase (F3H) mRNA, complete cds [*Paeonia lactiflora*] |
| *c92573_g1* | 769.63 | 717.46 | 0.04 | flavanone 3-hydroxylase [*Paeonia suffruticosa*] |
| | | *F3'H* | | |
| *c98915_g1* | 79.78 | 30.05 | 0.94 | flavonoid 3'-hydroxylase (F3'H) mRNA, complete cds [*Paeonia lactiflora*] |
| | | *DFR* | | |
| *c74115_g1* | 8.50 | 7.52 | 0.11 | Bifunctional dihydroflavonol 4-reductase/flavanone 4-reductase [*Malus domestica*] |
| *c84488_g1* | 109.11 | 100.05 | 0.06 | Bifunctional dihydroflavonol 4-reductase/flavanone 4-reductase [*Morus notabilis*] |
| *c88288_g1* | 1028.27 | 1199.37 | -0.26 | dihydroflavonol 4-reductase [*Paeonia lactiflora*] |
| *c96059_g1* | 56.91 | 48.64 | 0.17 | Bifunctional dihydroflavonol 4-reductase/flavanone 4-reductase isoform 1 [*Theobroma cacao*] |
| | | *ANS* | | |
| *c92767_g1* | 356.44 | 312.32 | 0.12 | anthocyanidin synthase (ANS) mRNA, complete cds [*Paeonia lactiflora*] |
| | | *FLS* | | |
| *c62726_g1* | 4.48 | 0.98 | 1.87 | flavonol synthase, putative, mRNA [*Ricinus communis*] |
| *c62726_g2* | 5.92 | 1.13 | 2.00 | flavonol synthase, putative, mRNA [*Ricinus communis*] |
| *c97570_g1* | 4.06 | 4.10 | -0.07 | flavonol synthase, partial [*Paeonia lactiflora*] |
| | | *FLS/F3H* | | |
| *c66711_g2* | 0.42 | 0.17 | 0.35 | Flavonol synthase/flavanone 3-hydroxylase, putative [*Ricinus communis*] |
| *c102156_g2* | 16.03 | 10.66 | 0.48 | Flavonol synthase/flavanone 3-hydroxylase, putative, mRNA [*Ricinus communis*] |

| | | | | |
|---|---|---|---|---|
| *c90288_g1* | 1.09 | 0.11 | 1.98 | PREDICTED: flavonol synthase/flavanone 3-hydroxylase-like [*Gossypium raimondii*] |
| *c90288_g2* | 2.06 | 0.55 | 1.45 | PREDICTED: flavonol synthase/flavanone 3-hydroxylase-like [*Eucalyptus grandis*] |
| *c74235_g1* | 0.42 | 0.25 | 0.47 | PREDICTED: flavonol synthase/flavanone 3-hydroxylase-like [*Jatropha curcas*] |
| *c95301_g1* | 155.63 | 295.90 | -0.96 | PREDICTED: flavonol synthase/flavanone 3-hydroxylase-like [*Citrus sinensis*] |
| | | | | *UAGT* |
| *c95107_g1* | 13.04 | 51.44 | -1.98 | PREDICTED: anthocyanidin 3-*O*-glucosyltransferase 2-like [*Prunus mume*] |
| *c128309_g1* | 0.17 | 13.39 | -1.39 | PREDICTED: anthocyanidin 3-*O*-glucosyltransferase 2 (LOC100248109), mRNA [*Vitis vinifera*] |
| *c8473_g1* | 74.18 | 123.05 | -0.76 | PREDICTED: anthocyanidin 3-*O*-glucosyltransferase 2-like [*Populus euphratica*] |
| *c99617_g1* | 244.51 | 363.01 | -0.60 | Anthocyanidin 3-*O*-glucosyltransferase [*Vitis vinifera*] |
| *c44319_g1* | 12.20 | 16.21 | -0.46 | Anthocyanidin 5,3-*O*-glucosyltransferase [*Rosa hybrid*] |
| *c51545_g1* | 0.14 | 0.19 | -0.24 | PREDICTED: anthocyanidin 3-*O*-glucosyltransferase 2-like [*Prunus mume*] |
| *c44249_g1* | 0.08 | 0.16 | -0.20 | Anthocyanidin 5,3-*O*-glucosyltransferase [*Rosa hybrid*] |
| *c93571_g1* | 3.72 | 4.08 | -0.17 | Anthocyanidin 5,3-*O*-glucosyltransferase [*Rosa hybrid*] |
| *c96046_g2* | 52.38 | 56.40 | -0.16 | Anthocyanidin-3-*O*-glucoside rhamnosyltransferase [*Petunia hybrida*] |
| *c132695_g1* | 0.10 | 0.12 | -0.05 | PREDICTED: anthocyanidin 3-*O*-glucosyltransferase 2-like [*Populus euphratica*] |
| *c126517_g1* | 0.31 | 0.31 | -0.02 | PREDICTED: anthocyanidin 3-*O*-glucosyltransferase 5-like [*Jatropha curcas*] |
| *c9624_g1* | 0.14 | 0.10 | 0.07 | Anthocyanidin 3-*O*-glucosyltransferase [*Solanum melongena*] |
| *c97667_g1* | 8.01 | 6.70 | 0.19 | PREDICTED: anthocyanidin 3-*O*-glucosyltransferase 2-like [*Eucalyptus grandis*] |
| *c51545_g2* | 0.26 | 0.12 | 0.29 | PREDICTED: anthocyanidin 3-*O*-glucosyltransferase 2-like [*Nelumbo nucifera*] |
| *c93787_g2* | 2.11 | 1.53 | 0.32 | PREDICTED: anthocyanidin 5,3-*O*-glucosyltransferase-like, partial [*Vitis vinifera*] |
| *c95696_g1* | 17.48 | 2.55 | 1.74 | PREDICTED: anthocyanidin 3-*O*-glucosyltransferase 5-like [*Fragaria vesca* subsp. *vesca*] |
| *c95696_g2* | 15.68 | 3.59 | 1.92 | PREDICTED: anthocyanidin 3-*O*-glucosyltransferase 5 [*Vitis vinifera*] |

**Tab. S3 The contact list between UDP-glucose with PhUGT78A22.**

| NO. | The atoms in UDP-glucose | The residues in PhUGT78A22 | Interaction type |
|---|---|---|---|
| 1 | O5 | Glu357.OE1 | Hydrogen bond interaction |
| 2 | O5 | Glu357.OE2 | Hydrogen bond interaction |
| 3 | O6 | Glu357.OE2 | Hydrogen bond interaction |
| 4 | O8 | Asp373.OD2 | Hydrogen bond interaction |
| 5 | O11 | Asp373.OD1 | Hydrogen bond interaction |
| 6 | N21 | Ala332.O | Hydrogen bond interaction |
| 7 | O10 | Thr19.OG1 | Hydrogen bond interaction |
| 8 | O18 | Gly278.CA | Hydrogen bond interaction |
| 9 | O18 | Thr279.N | Hydrogen bond interaction |
| 10 | O18 | Thr279.OG1 | Hydrogen bond interaction |
| 11 | O18 | His349.NE2 | Hydrogen bond interaction |
| 12 | O19 | Ser305.OG | Hydrogen bond interaction |
| 13 | O19 | Ala332.N | Hydrogen bond interaction |
| 14 | benzene ring | Trp331 | PI-PI interaction |

**Tab. S4 The contact list between Cy, Cy3G, Pn and Pn3G with PhUGT78A22 + UDP-glucose.**

| Cy | | | |
|---|---|---|---|
| NO. | The atoms in Cy | The residues in PhUGT78A22 | Interaction type |
| 1 | O5 | His20.NE2 | Hydrogen bond interaction |
| 2 | O3 | Glu186.O | Hydrogen bond interaction |
| 3 | benzene ring | Phe118.CZ | Pi-H interaction |
| 4 | pyran | Gly372.CA | Pi-H interaction |

| Cy3G | | | |
|---|---|---|---|
| NO. | The atoms in Cy3G | The residues in PhUGT78A22 | Interaction type |
| 1 | O3 | Gln81.OE1 | Hydrogen bond interaction |
| 2 | O4 | Gln81.OE1 | Hydrogen bond interaction |
| 3 | O8 | Ala280.O | Hydrogen bond interaction |
| 4 | O10 | Glu186.O | Hydrogen bond interaction |
| 5 | O10 | His147.NE2 | Hydrogen bond interaction |
| 6 | benzene ring | Phe371.benzene ring | Pi-Pi interaction |

| Pn | | | |
|---|---|---|---|
| NO. | The atoms in Pn | The residues in PhUGT78A22 | Interaction type |
| 1 | O3 | Glu186.O | Hydrogen bond interaction |
| 2 | O1 | Asp373.OD1 | Salt bond |
| 3 | Benzene ring | Phe118.CZ | Pi-H interaction |
| 4 | Pyran | Gly372.CA | Pi-H interaction |

| Pn3G | | | |
|---|---|---|---|
| NO. | The atoms in Pn3G | The residues in PhUGT78A22 | Interaction type |
| 1 | O3 | Ala280.O | Hydrogen bond interaction |
| 2 | O8 | Glu186.O | Hydrogen bond interaction |
| 3 | O11 | Thr279.OG1 | Hydrogen bond interaction |
| 4 | O9 | Gln374.NE2 | Hydrogen bond interaction |
| 5 | O7 | Asp373.OD1 | Salt bond |
| 6 | O5 | Phe371 Benzene ring | Pi-H interaction |
| 7 | Benzene ring | Phe118.CZ | Pi-H interaction |

**Tab. S5 The primers used in this study.**

| | Forward primer (5'→ 3') | Reverse primer (5'→ 3') |
|---|---|---|
| | For quantitative RT-PCR analysis | |
| *Poβ-TUB* | GCACCAAAGAAGTGGACGAACAAAT | AGTAAACTGTTCACTCACACGCCTG |
| *PhUGT78A22* | TTATCACAAGAAAAGGGAAAGAAG | AAAACACAATTACAAACCAAAACAT |
| *c95107_g1/UAGT* | GTGATGGGGAGATAAGAAAGAGGGT | ATTCATTACAAAGAATTACAACGAA |
| *c128309_g1/UAGT* | AGGAAATGAAGGAAATCAGCAAAAA | TGAGATAAAATAAAAATACCAAACG |
| *c8473_g1/UAGT* | TTGAAACATACAATGAGCAAAGTT | TTGGGTTGAGGGAGGGTGACGAAA |
| *c95696_g1/UAGT* | AGGGCGAGAGAATTACAAAGAAG | AATACAACAAAGGATAGCCAAAC |
| *c95696_g2/UAGT* | GGAAAGCCAACACCAATAACAAAAT | AATACAACAAAGGATAGCCAAACAT |
| | For subcellular localization | |
| Super promoter:: *PhUGT78A22-GFP* | ctgcaggggcccggggtcgacATGACGTCGATGACTAACGAGC | ccatggtaccggatccactagtAGCAATATCCTTATGCCTTGATAC |
| | For *in vitro* enzymatic assays | |
| pGEX4T-2-*PhUGT78A22* | gatctggttccgcgtggatccATGACGTCGATGACTAACGAGCC | ctcgagtcgacccgggaattcTTAAGCAATATCCTTATGCCTTGATAC |
| | For VIGS | |
| TRV2::*PhUGT78A22* | gtgagtaaggttaccgaattcGGGGAGATCAGAGGGTTGAAA | cgtgagctcggtaccggatccTTACCATAATAAAACAATACAAAAGGGTAC |
